# Systematic screen uncovers regulator contributions to chemical cues in *Escherichia coli*

**DOI:** 10.1371/journal.pbio.3003260

**Published:** 2025-07-22

**Authors:** Christoph Binsfeld, Roberto Olayo-Alarcon, Lucía Pérez Jiménez, Morgane Wartel, Mara Stadler, André Mateus, Christian Müller, Ana Rita Brochado

**Affiliations:** 1 Department of Microbiology, Biocenter, University of Würzburg, Würzburg, Germany; 2 Department of Statistics, Ludwig-Maximilians-Universität München, München, Germany; 3 Institute of Computational Biology, Helmholtz Zentrum München, München, Germany; 4 Department of Molecular Biology, Umeå University, Umeå, Sweden; 5 Department of Chemistry, Umeå University, Umeå, Sweden; 6 Genome Biology Unit, European Molecular Biology Laboratory, Heidelberg, Germany; 7 The Laboratory for Molecular Infection Medicine Sweden (MIMS), Umeå, Sweden; 8 Center for Computational Mathematics, Flatiron Institute, New York, New York, United States of America; 9 Interfaculty Institute of Microbiology & Infection Medicine Tübingen (IMIT), University of Tübingen, Tübingen, Germany; 10 Cluster of Excellence ‘Controlling Microbes to Fight Infections’ (CMFI), University of Tübingen, Tübingen, Germany; Max Planck Institute for Terrestrial Microbiology: Max-Planck-Institut fur terrestrische Mikrobiologie, GERMANY

## Abstract

In Gram-negative bacteria, the uptake and export of a wide range of molecules, including antibiotics, is facilitated by porins and efflux pumps. Because of their role in regulating small molecule permeability of the outer and inner membrane, these transport machineries are tightly regulated at the transcriptional and post-transcriptional levels. However, regulation of transport by external chemical cues remains poorly understood. Here we investigated transcriptional regulation of three prominent transporter genes in *Escherichia coli* across 94 defined chemical cues, and simultaneously mapped the contributions of the key regulators MarA, SoxS and Rob to promoter activity. One third of all tested compounds triggered transcriptional changes, the majority of which were previously unknown. Importantly, we exposed main drivers of transport control in *E. coli*, e.g., bacteriostatic but not bactericidal antibiotics trigger the expression of efflux pumps, and Rob contributes to ~1/3 of all measured transcriptional changes, thereby emerging as a more prominent regulator of transport than previously thought. We showcase the potential of our resource by elucidating the molecular mechanism of antibiotic antagonisms with widely consumed caffeine in *E. coli*. Altogether, our analysis provides a quantitative overview of how different regulators orchestrate the transcriptional response of major transport determinants to environmental chemical cues.

## Abstract 2

Transport machineries are crucial for antibiotic resistance, but their regulation by external cues is poorly understood. This study identifies environmental chemicals that regulate transporter transcription, identifying the transcription factor Rob as a key modulator of efflux pump expression in E. coli.

## Introduction

Gram-negative bacteria have a double membrane surrounding their cell wall, which acts as a selective barrier against the environment. Influx and efflux of chemically diverse small molecules, including harmful substances, but also nutrients and intracellular metabolites, across the cell envelope is facilitated by protein-channels, namely outer membrane porins (OMPs) and efflux pumps [1,2]. A delicate balance between porin-mediated passive uptake and a fast active efflux imposes a permeability barrier, and it is therefore a crucial determinant for bacteria to thrive in harsh environments. Numerous mutations in efflux pumps, porins and their regulatory elements have been associated with antibiotic resistance in clinical isolates [2–5]. Furthermore, pump deletion mutants have impaired host-intracellular survival [6] and restricted antibiotic persistence phenotypes [7–9]. It is also becoming increasingly clear that the same import/export machineries are determinants for sensitivity to non-antibiotic drugs, not only in pathogenic bacteria but also in commensal members of bacterial communities, such as the gut microbiota [10–12]. Our own previous work suggests that transport regulation is a determinant of synergy and antagonism in bacteria, at least as important as the antibiotic targets themselves [13,14]. Yet, our knowledge of how bacteria control their import/export machineries across environments remains limited, preventing better design of treatment strategies.

**Fig 1:**
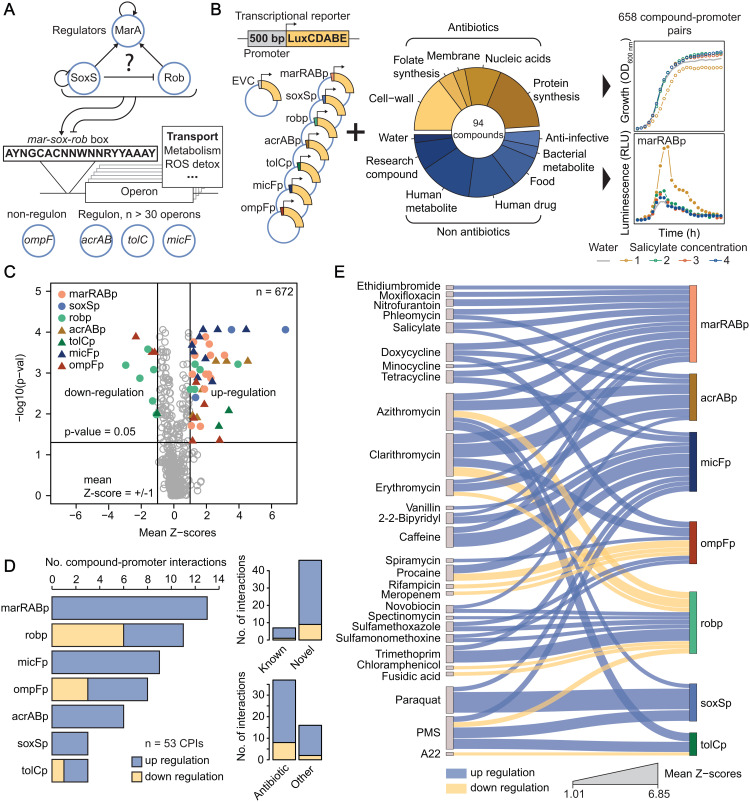
Unravelling transcriptional regulation of drug transport-related genes in *E. coli* under chemical stress. (A) Simplified schematic representation of the mar-sox-rob network, as well as placement of the 7 promoters selected for this study. Previously described auto- and cross-regulation between the three regulators [2,41], as well as their degenerate binding sequence (mar-sox-rob box [76]) are also represented. (B) Schematic overview of the screening approach. Lux-based transcriptional reporters of 7 key transport-related genes and the compound library used in this study to probe 658 compound-promoter interactions. Growth and luminescence were periodically measured over 12 h. (C) Compound-promoter interactions in *E. coli*. Volcano plot summarizing the screen results shows 53 significant CPIs (colored by promoter) amongst 658 tested (+water, n = 672 in total). X-axis: mean Z-scores (n = 4 concentrations x 2 biological replicates = 8). Y-axis: Benjamini Hochberg adjusted p-value of double-sided rank-sum statistical test between Z-scores of compound-promoter pairs (n = 8) and water (n = 16). (D) General features of compound-promoter interactions (CPIs). Number (No) of interactions per promoter (left), their classification according to novelty (upper right corner) and whether they involve an antibiotic (lower right corner) are shown. (E) CPI network. Fifty-three significant compound-promoter interactions are shown as edges in a Sankey diagram connecting the compounds (left, source nodes) to the promoters (right, target nodes). Edge thickness represents mean Z-scores (n = 8), while node size represents the total number of interactions. The underlying data for all panels can be found in S7 Table.

Three major proteins, including OmpF, represent a substantial fraction of the total number of outer membrane porins in *E. coli*. Together, they facilitate influx of a wide range of anionic and cationic small molecules, including clinically important antibiotics, such as β-lactams and fluoroquinolones [5]. In regard to efflux, six efflux pump families have been described in bacteria, using either ATP or electrochemical gradients as energy source for active transport. The resistance-nodulation-cell division (RND) pump AcrAB-TolC is among the most well-characterized efflux pumps in *Enterobacteriaceae*, including *E. coli* [2]. This is a tri-partite protein complex spanning the inner- and outer-membrane and effluxing a wide range of antibiotics [2]. Due to their fundamental role in controlling membrane permeability, influx and efflux machineries are highly regulated at transcriptional and post-transcriptional levels [2]. Regulatory mechanisms include two-component systems (e.g., CpxAR), small proteins that modulate pump specificity (e.g., AcrZ), and global transcriptional regulators such as the transcription factors MarA, SoxS, Rob and RamA (latter absent in *E. coli*). MarA, SoxS and Rob bind the so called mar-sox-rob box, a degenerate sequence of ~20 base-pairs found in multiple promoter sequences of over 40 genes in *E. coli*, including *acrAB, tolC* and *micF,* which encodes *a* small RNA involved in porin regulation (Fig 1A) [15]. This arrangement enables *E. coli* to orchestrate a complex transcriptional network in response to different environmental cues, such as oxidative stress, toxic compounds, acidic pH, bile acids and more [2,15–18]. Extensive efforts have been directed to structurally and functionally characterize several components of this network, including transcriptome analysis [19–22], identification of regulator binding sites using chromatin immunoprecipitation and DNA sequencing [23], identification of canonical chemical cues [16–18,24,25], ligand-regulator structure determination [26,27], pumps and porins specificity studies [28,29], among many others. Efflux pump inhibitors, ideal compounds for potentiating antibiotic activity by directly impairing efflux, have been identified [30]. However, given the complexity, redundancy and extensive cross-talk inherent to this regulatory network [2,15] (Fig 1A), current knowledge is still not sufficient to enable full understanding of network operational modes, namely whether and how the hierarchy of regulator-responsive promoters changes across environmental cues.

Here we propose a systematic and quantitative approach to elucidate transcriptional control of 7 key transport-related genes in the model organism *E. coli* across 94 chemically defined environments. Our results expose several new modulators of bacterial transport transcriptional control, such as the macrolide antibiotics, but also non-antibiotic compounds such as caffeine. In a complementary experimental approach using regulator deletion mutants, we quantified regulator contribution to promoter activity under all 94 compounds using a simple regression model, and mapped how the same regulators can promote or hinder transcriptional regulation of a target gene depending on the chemical cue. We capitalize on our unique systematic approach to uncover general drivers of transport regulation in *E. coli*, for instance Rob, which appears to have a much more prevalent role in transcriptional control of transport than previously acknowledged. Our findings illustrate how a comprehensive approach is crucial for revealing operational modes of highly complex regulatory networks, such as the mar-sox-rob network. Finally, we showcase the mechanistic potential of our approach by elucidating the molecular mechanism by which caffeine, a widely consumed food ingredient, induces Rob-dependent low level resistance to fluroquinolones and β-lactams in *E. coli*, via decreased uptake linked to extensive modulation of outer-membrane protein levels.

## Results

### Unravelling transcriptional regulation of transport-related genes in *E. coli* under chemical stress

We set out to systematically investigate the transcriptional response of a set of prominent genes controlling transport in *E. coli* across 94 defined chemical stresses in a concentration resolved manner. To meet our aim of uncovering the cross-talk between transport-related genes, rather than genome-wide transcriptional analysis, we prioritized an experimental design that enables close quantitative monitoring of transcriptional changes (even if small) in a set of promoters over a range of compound concentrations. Therefore, we constructed plasmid-based luminescence reporters for 7 genes, 6 of which contain the degenerate mar-sox-rob box in their promoter region (Fig 1A): the key transcription factors *marA*, *soxS* and *rob*, *acrAB* and *tolC*, which are both components of the major efflux pump AcrAB-TolC, and the small-RNA MicF, a post-transcriptional regulator of *ompF* [31]. In addition, we included a reporter of *ompF* transcription, to monitor the transcriptional response of transport-related genes that are not under direct control of MarA, SoxS or Rob (Materials and methods, S1 Table). Our promoter selection was prioritized based on impact of each gene product on intrinsic antibiotic resistance. Even though *E. coli* has more than 30 efflux pumps, AcrAB-TolC is widely described as the most impactful for intrinsic antibiotic resistance, and, in addition, it is under tight transcriptional control [28,32,33]. Similarly, OmpF is the dominant porin for intrinsic resistance under our working conditions [29,34], and mutations in *acrB* and *ompF* are frequently selected in laboratory evolution experiments under antibiotic pressure, highlighting their determinant role in intrinsic resistance [35]. MicF was included because of its prominent role in OmpF posttranscriptional control while being itself under tight transcriptional regulation [31]. Finally, MarA, SoxS and Rob were included because they orchestrate large transcriptional responses to different environmental and chemical stresses, and their regulons include several of the transport genes mentioned above. In total, we probed 658 compound-promoter pairs. As many compounds, and specifically antibiotics, are known to cause vast transcriptional effects [36,37], we included a reporter strain containing a promoterless/leaky luminescence reporter (empty vector control, EVC) to better control for possible non-specific transcriptional effects of each compound. We assembled a collection containing 94 compounds including all major antibiotic classes, human-targeted drugs (e.g., aspirin), gut metabolites (e.g., bile acids) and small-molecules found in common foods (e.g., vanillin, Fig 1B and S2 Table). We ensured high overlap of the compound library with our previous work on systematic assessment of drug combinations in Gram-negative bacteria (61 out of 79 compounds) [13], to enable downstream integration of the datasets for interpretation purposes. All compound-promoter pairs were probed in duplicates across four compound concentrations (2-fold dilutions, S1A Fig). Maximum concentrations were adjusted to be close to minimum inhibitory concentration (MIC) for antimicrobials, 500 µM for most non-antimicrobials, and up to 1 mM for small compounds with similarity to canonical inducers (positive controls, e.g., salicylate, S2 Table, Materials and methods). Briefly, growth (optical density, OD_600 nm_) and luminescence for each reporter strain were periodically monitored over 12 hours in the presence of each individual compound at all concentrations (Materials and methods). Area under the curve for a period of 8h (AUC) was used as a proxy for growth (OD_AUC_) and luminescence (Lux_AUC_) profiles (onset of stationary phase, S1B Fig and S3 Table). As expected, growth was reasonably constant across all reporter strains, while luminescence showed a large dynamic range depending on the promoter, with tolCp showing the lowest, and ompFp the highest signal (S1C and S1D Fig). We excluded the possibility that some compounds, namely protein synthesis inhibitors, could increase plasmid copy number (as previously reported for different origin of replication [38]) by quantitative PCR (S1E Fig). High data quality is reflected by Pearson correlation between replicates above 0.8 across all promoters, except for tolCp, likely due to its weakness (S1F Fig). Nonetheless, since the effect of compounds expected to trigger its expression via oxidative stress – e.g., phenazine methosulfate – could be captured here, we decided to keep tolCp in our dataset.

Next we systematically assessed *Compound-Promoter Interactions* (CPIs), which we define as increased or decreased promoter activity as measured by our luminescence reporter in the presence of the compound (Materials and methods, S1B Fig). Briefly, we computed an *interaction score* for any given compound-promoter pair based on its deviation of normalized luminescence (Lux_AUC_/OD_AUC_) from the c*ompound-EVC*. We subsequently Z-transformed the interaction scores (Z-scores) to allow comparability of promoters of varying signal intensity. Finally, significant CPIs were called based on a double cutoff on mean Z-scores and rank-sum test p-value comparing the Z-score distributions of each compound-promoter with that of water-promoter (Fig 1C, Materials and methods, S4 Table). We identified 53 CPIs distributed across all promoters and 28 out of the 94 compounds (S2A Fig), with induction being stronger and more prevalent than repression (43 versus 10 instances, Fig 1D). While the majority of the 28 compounds are antibiotics, ~ 1/3 of all identified CPIs involve non-antibiotic compounds, showing that non-antibiotics also modulate transport across the cell envelope [12,13] (Fig 1D). The number of interactions per promoter varies between three for soxSp and tolCp and 13 for marRABp, confirming differences in promoter specificity towards chemical cues. In addition, more than half of the 28 compounds triggered at least two promoters, confirming regulatory cross-talk between the major players of transport in *E. coli* (S2A Fig). Protein synthesis inhibitors emerge as the most promiscuous compounds, as tetracyclines and macrolides triggered simultaneous transcriptional responses in up to 5 out of 7 promoters (Fig 1E).

Looking at individual CPIs, we were able to recapitulate several canonical compound-promoter pairs, including salicylate*-*marRABp [18,24], paraquat*-*soxSp [17], procaine-micFp [31], procaine-ompFp [39], and 2,2-bipyridyl-micFp [25], confirming that our screen correctly captures known interactions (Fig 1E and examples at S2B Fig). Compound-dependent repression occurred mostly for the *rob* promoter, consistent with previous reports that *rob* is subject to repression by, e.g., MarA and SoxS [40–42] (Fig 1C, E). Furthermore, our network shows a rather specific oxidative-stress response of soxSp, also consistent with previous reports [41]. Importantly, our approach misses known interactions, for instance vanillin-marRABp or chloramphenicol-marRABp [13,43]. We attribute this to the facts that we used stringent statistical cutoffs to minimize false positive discovery, and comparatively low concentrations aiming at identifying stronger and specific interactions. Thus, we are most likely underestimating CPIs. Nevertheless, ~ 80% of all CPIs we describe here have not been previously reported (Fig 1D). For instance, we identified tetracycline derivatives, e.g., doxycycline and minocycline, as novel marRABp inducers, beyond the previously reported tetracycline [43]. Importantly, we identified macrolides (azithromycin, clarithromycin and erythromycin) and antifolates (sulfonamides and trimethoprim) as novel antibiotic classes triggering several of the tested promoters (Fig 1E). Beyond antibiotics, we identified the widely consumed food ingredient caffeine as novel marRABp and micFp inducer. Among new inducers, we independently validated that *marRAB* native expression is indeed induced by clarithromycin treatment using RTq-PCR (S2C Fig). In addition, RTq-PCR analysis revealed that also sulfamethoxazole triggers marRABp, although our screening approach was unable to capture it due to stringent cutoffs (S2C Fig).

### General principles driving transport compound-promoter interactions

**Fig 2:**
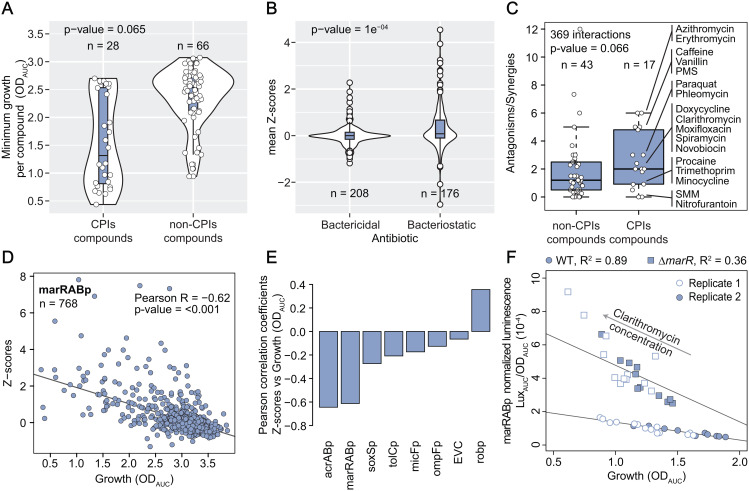
General principles driving transport compound-promoter interactions. (A) Most compounds identified within CPIs have antimicrobial effect. Distribution of minimum growth (0.1 quantile of all OD_AUC_ measurements for a given compound, 4 concentrations x 8 strains x 2 replicates = 64 values) of all compounds tested (n_total_ = 94), classified according to whether they are (or not) involved in CPIs. P-value from a double-sided rank-sum statistical test between the two distribution depicted in the plot is shown (null-hypothesis is that both distributions are identical). Boxplots indicate 25^th^, 50^th^ and 75^th^ percentiles, and whiskers extend up to 1.5 times the interquartile range (IQR) from the 25^th^ and 75^th^ percentiles. (B) Bacteriostatic antibiotics are over-represented within strong CPIs. Mean Z-scores distributions of all tested compound-promoter pairs involving antibiotics (n_total_ = 384), classified according to whether the antibiotic is bactericidal or bacteriostatic. P-value from a double-sided rank-sum statistical test between the two distributions depicted in the plot is shown (null-hypothesis is that both distributions are identical). Boxplots indicate 25^th^, 50^th^ and 75^th^ percentiles, and whiskers extend up to 1.5 x IQR from the 25^th^ and 75^th^ percentiles. (C) Compounds within CPIs are over-represented among antagonistic drug interactions. Boxplots of ratios antagonisms over synergies (total 369 interactions) for all tested compounds which overlap with our previous work [13] (n_total_ = 60), classified according to whether they are (or not) involved in CPIs. p-value from a one-sided rank-sum statistical test shown. Center, upper and bottom lines represent 25^th^, 50^th^ and 75^th^ percentiles, whiskers extend to 1.5x IQR and points beyond whiskers are represented individually. **(D)** marRABp activity inversely correlates with growth. Z-scores of all compound-marRABp tested pairs including water across 4 concentrations and 2 biological replicates (n) are plotted against growth (OD_AUC_). A strong negative linear relationship is illustrated by the line of best fit (Huber robust model). Correlation p-value (double sided **t* test*) shown. (E) Promoter activity is generally not correlated with growth. Pearson correlation coefficients of Z-scores vs. growth (OD_AUC_) for each individual promoter. A strong negative Pearson correlation (>0.6) is only observed for marRABp and acrABp, while robp tends to show the opposite behavior. Correlation p-value (double sided *t *t*est*) < 0.005 for all promoters. (F) Induction of marRABp by clarithromycin, as well as its negative correlation with growth remain, irrespective of the presence of MarR. Luminescence profiles over growth were measured across a linear range of clarithromycin concentrations from 0 µg/ml to 119.6 µg/ml in wild-type and ∆*marR* background. Growth-normalized luminescence is plotted against growth for two independent biological replicates, and lines-of-best-fit are shown to highlight strong correlation between the two variables. The underlying data for all panels can be found in S7 Table.

Capitalizing on our efforts of assessing promoter activity across environmental cues, we next aimed at uncovering general features driving environment-dependent transport regulation in *E. coli*. Since many of our CPIs involved antibiotics, we questioned whether antimicrobial activity is a pre-requisite for modulating gene expression. Indeed, we observed that our set of 28 compounds instigate, on average, lower minimum growth (OD_AUC_) when compared to the compounds that did not trigger the tested promoters (Fig 2A). However, also compounds without antimicrobial activity at the concentrations tested (e.g., vanillin, caffeine, growth identical to control at all concentrations tested) are represented among our CPIs, and thus antimicrobial activity is not required to trigger transcriptional changes in transport related genes (Fig 2A). Next, we asked whether the nature of the antibiotic – bactericidal or bacteriostatic – correlates with its ability to trigger transcriptional changes amid the tested promoters. Interestingly, we found that bacteriostatic antibiotics are enriched for extreme Z-scores (Fig 2B). In fact, bacteriostatic antibiotics such as tetracyclines, macrolides or sulfonamides account for half of the 53 CPIs described here. Next, because our previous work showed that antagonism is often associated with decreased intracellular antibiotic concentrations [13], we hypothesized that compounds impacting transport regulation could decrease the concentration of certain antibiotics, and thus exhibit antibiotic antagonism. We combined our results with the data from our previous study, and found that the compounds that are represented in our set of 53 CPIs have indeed a higher chance than other tested compounds to be involved in antagonistic interactions (Fig 2C, p-value = 0.066).

Driven by the fact that growth inhibition is a strong trait of the compounds represented in the 53 CPIs (Fig 2A), we probed whether growth alone could explain the extent of promoter induction for any given promoter. By assessing correlation of Z-scores versus growth (OD_AUC_) across all compounds for each individual promoter, we observed strong (Pearson R > 0.6) and significant negative correlation for marRABp and acrABp – meaning that these promoters get progressively stronger activation with increasing inhibitory capacity of the compound at hand (Figs 2D, E and S2D). This striking observation points towards a generalizable, non-specific, growth-driven response of marRABp across environments, in addition to the widely reported stress-specific response based on de-repression of promoter activity through compound-repressor binding (salicylate-MarR) [26,44]. This finding provides a basis for promiscuous marRABp transcriptional activation by structurally diverse compounds, such as salicylate, clarithromycin and sulfamethoxazole (S2E Fig). We then confirmed that concentration-dependent transcriptional activation of marRABp by clarithromycin and sulfamethoxazole remains irrespective of whether its repressor MarR is present or not (Figs 2F and S2F). Even though weaker, the inverse tendency was observed for robp, where reporter expression increases with growth (S2D Fig). This finding increases the scope of previous observations that MarA may directly or indirectly cause *rob* down-regulation [40,41], as we find they follow this opposite trend across several environmental conditions. For the remaining promoters no comparable correlation was observed, suggesting growth-independent regulation.

### Mapping regulator contributions to compound-promoter interactions

**Fig 3:**
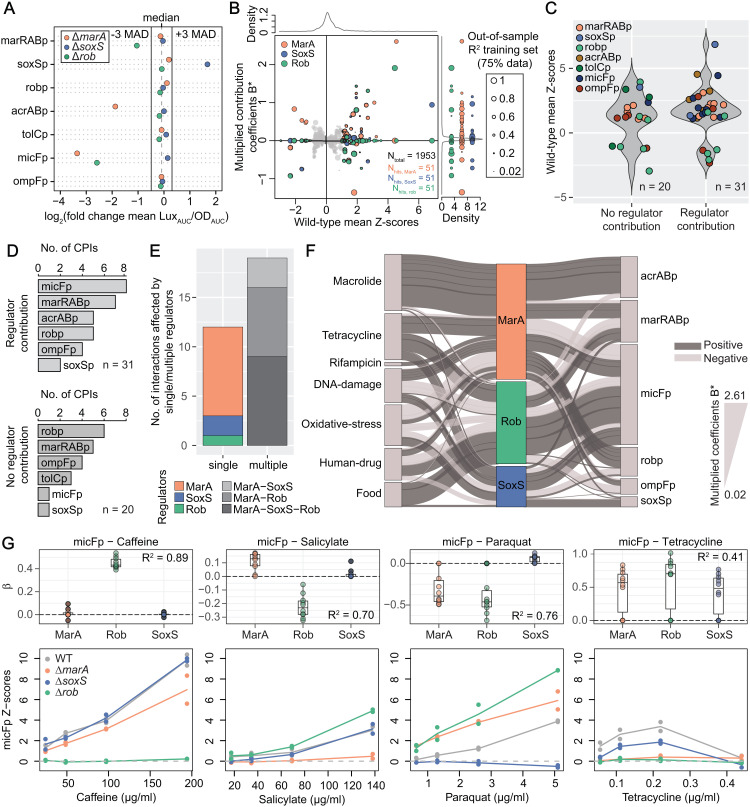
Mapping regulator contributions to compound-promoter interactions. (A) Deletion of *marA*, *soxS* and *rob* alter promoter basal activity. log_2_ fold-change of water-promoter mean normalized luminescence (Lux_AUC_/OD_AUC_) of each deletion background in relation to the wild-type is plotted. The dashed line represents the median of log_2_ fold-change of water-promoter mean Lux_AUC_/OD_AUC_ across all promoters and deletion backgrounds. Full lines show + /-3 MAD (median absolute deviation). Water-promoter mean Lux_AUC_/OD_AUC_ over 16 replicates per reporter (n = 8 x 2 biological replicates = 16). (B) Regulator contributions to CPIs are complex and multi-directional. Multiplied coefficients (B*) of MarA, SoxS and Rob of 651 compound-promoter pairs vs. wild type mean Z-scores are plotted (n_total_). Dot size reflects the out-of-sample R^2^ of the corresponding compound-promoter pair. Pairs without R^2^ are represented with the smallest size. Density distributions of total B* and wild-type mean Z-scores are represented on the top and right side of the main plot, respectively. B* corresponding to 51 significant CPIs are colored according to regulator and projected into the right axis to facilitate visualization. (C) Most CPIs feature contributions of at least one regulator. Mean Z-score distributions of 51 CPIs (colored by promoter) classified on whether (or not) they have at least one non-zero B*. (D) Almost all acrABp and micFp CPIs depend on MarA, SoxS or Rob. Number of CPIs with (B* ≠ 0, upper plot) and without (B* = 0, bottom plot) regulator contributions distributed by promoter. (E) Most CPIs depend on two or all three regulators. Number of CPIs depending on single of multiple regulators, colored according to which regulators have B* ≠ 0. (F) Regulator-CPI network. Regulator contributions to 31 CPIs are shown as edges in a Sankey diagram connecting the compounds (left nodes, grouped according to class or purpose) the promoters (right nodes) via the regulators (middle). Edge thickness and node size represent B*, and the total number of interactions, respectively. (G) Regulator contributions to specific promoters are compound dependent. β (top) and Z-scores (bottom) of micFp interactions with caffeine, salicylate, paraquat and tetracycline in wild-type and ∆*marA*, ∆*soxS* and ∆*rob*. β corresponds to the regression coefficients quantifying the contribution of each regulator to the observed effect. Boxplots with β from the 10-fold cross validation are shown - center, upper and bottom lines represent 25^th^, 50^th^ and 75^th^ percentiles, whiskers extend to 1.5x IQR and points beyond whiskers are represented individually. Lines are colored by strain and indicate mean Z-scores of two biological replicates (dots). Depending on the compound, micFp activity mostly depends on a single (caffeine), on two (salicylate), or on all three regulators (tetracycline and paraquat). The underlying data for all panels can be found in S7 Table.

To gain more specific insight into the mar-sox-rob network operational modes – in particular into whether and how regulator-promoter hierarchy changes across environmental cues – we generated individual knockout strains of the regulators *marA*, *soxS* and *rob*, and again probed the 658 compound-promoter pairs with our initial chemical library. Promoter preference towards a given regulator (as seen by loss of reporter activity upon regulator deletion) could already be observed for marRABp, soxSp, acrABp and micFp through altered basal promoter activity without any stress (Fig 3A). In most cases there seems to be a preference for a single regulator, for instance, acrABp activity decreases only when MarA is absent. Nonetheless, micFp seems to be differently controlled, as its activity decreases in the absence of either MarA or Rob, pointing towards cooperation between these two regulators to sustain *micF* basal expression. Our findings add on previous observations of how the different regulators influence promoter activity [41,45]. For instance, MarA not only increases acrABp and micFp activity upon overexpression as it has been shown before [46,47], but also determines their basal expression in the absence of any chemical or genetic stress (Fig 3A). In addition, we observed that deletion of either *marA*, *soxS* or *rob* sensitizes *E. coli* towards multiple growth inhibiting compounds, particularly at sub-MIC concentrations (S3A Fig), This highlights that, despite their different responses to various compounds, the three regulators uniquely contribute to ensure optimal reaction to stress.

Next, we built a simple statistical model using Lasso regression for hierarchical interactions [48] to estimate the contribution of MarA, SoxS and/or Rob to CPIs in our dataset. Briefly, we modeled the transcriptional effect of each compound on a given promoter as a function of compound concentration and regulator presence/absence. The individual regulator and compound concentration contributions to the observed changes in promoter activity are captured in the model coefficients $\beta_{j}$ with j∈{conc,rob,marA,soxS} (Materials and methods, S3B Fig). Lasso penalization [49] was used to obtain sparse model coefficients, and as a result, the majority of the values for each $\beta_{j}$ across all modelled CPIs is 0 (or very nearly 0), with positive and negative deviations reflecting positive or negative contributions to promoter activity in the presence of a given compound (S3C Fig and S5 Table). As our aim was to build an interpretable statistical model, we used all of the available data for each CPI to estimate the corresponding regulator contributions $\beta_{j}$. To assess the robustness of these inferred contributions, we performed 10-fold cross-validation and calculated the average out-of-sample R² value (Materials and methods). Higher R² values indicate greater robustness and predictive reliability of the estimated regulator contributions. Firstly, the model accurately captures strong compound concentration dependent effects (reflected by large absolute values for $\beta_{\text{conc}}$, S3C Fig), stressing the added-value of concentration-resolved experiments. We also estimated coefficients for synergistic regulator contributions θ: θ_marA,soxS_, θ_marA,rob_ and θ_soxS,rob_. However, we observed that these are much more modest than single regulator coefficients (S3C Fig), and therefore decided to focus on single regulator contributions (β_*marA*_, β_*soxS*_, β_*rob*_) to the 51 CPIs identified in our initial wild-type dataset (2 CPIs, Phleomycin-marRABp and Phleomycin-acrABp, could not be assessed in the regulator deletion mutants due to very poor growth). As expected, compound promoter pairs with high interaction Z-scores tend to have higher out-of-sample R^2^ (S3D Fig), and significant CPIs have indeed higher out-of-sample R^2^ than the majority of all pairs (S3E Fig, two-sided rank-sum test p-value < 0.0001). In order to facilitate interpretation, we computed *multiplied model coefficients* (B*) by multiplying β by the absolute Z-score of the corresponding CPIs in the wild-type. Thus, B* reflect the overall relevance of a regulator towards change in promoter activity by a given compound in the wild-type, since it accounts for the amplitude of the change. B* differ from β in that all B* for weak (or null) compound-promoter interactions scores in the wild type are close to zero (Figs 3B and S3F). Notably, all regulators were found to have both positive and negative contributions to promoter activity depending on the compound-promoter pair (Fig 3B), suggesting a high network cross-talk and plasticity towards the environment. Consistent with its rather specific role in response to oxidative stress, SoxS has the tightest zero-centered coefficient B* distribution, with the least number of strong negative or positive contributions. MarA emerges as the most prominent regulator across all stresses, with the highest number of non-zero contributions, which are positive in most instances (Fig 3B). Interestingly, the far less well characterized Rob plays a more prominent role than SoxS across a variety of stresses, with both positive and negative strong contributions to ~1/3 of all CPIs (Fig 3B).

Overall, our approach captures contributions of MarA, SoxS or Rob to only 31 out of 51 compound-regulator pairs (Fig 3C, D). This result is expected, since contributions of other regulatory elements are certainly in place and not taken into account here – e.g., regulation by other transcription factors, such as OmpR or AcrR [2]. Concomitantly, several CPIs involving ompFp and robp – the first does not even contain a mar-sox-rob box – are not influenced by any of the three tested regulators (Fig 3D). Yet, we quantified MarA, SoxS or Rob contributions to as many interactions involving ompFp, presumably emerging from indirect regulatory network effects. Importantly, the three regulators play a role in pretty much all CPIs involving acrABp and micFp. Among the 31 CPIs for which we could map regulator contributions, only 12 mapped to a single regulator – MarA, SoxS or Rob (Fig 3E). For the remaining 19 CPIs, they can only be fully achieved when two or all three regulators are in place (Fig 3E). A more explicit/functional inspection of regulator contributions to CPIs revealed striking observations. First, the transport-controlling regulatory response to tetracyclines and macrolides is vastly different, despite their common mechanism of action – inhibition of protein synthesis (Fig 3F). While response to macrolides seems to be strictly driven by MarA, tetracyclines induce a much more complex response involving all three regulators. Another interesting observation is that acrABp expression is almost exclusively controlled by MarA across all compounds tested, with Rob playing a very minor role (Figs 3F and S3G, H). Even though MarA control of acrABp is well supported by several studies [46,50,51], our results indicate that this is a prevalent regulatory relationship across environments, where SoxS and Rob have minor and rather specific contributions. Interestingly, not all compounds triggering marRABp necessarily trigger acrABp though, so other factors, such as alternative regulation by AcrR might be determinant in these cases. A very different pattern is observed for micFp, where CPIs are the net outcome of a complex regulatory pattern of positive and negative contributions of MarA, SoxS and Rob (Fig 3F). We highlight a few examples where micFp expression is strongly influenced by one (caffeine), two (salicylate) or all three (tetracycline and paraquat) regulators (Fig 3F). Our results so far show how *E. coli* diversifies its response to different chemical stresses and, most importantly, provide a quantitative overview on regulator contribution to promoter activity.

### Caffeine induces proteome-wide changes in a Rob-dependent manner

**Fig 4:**
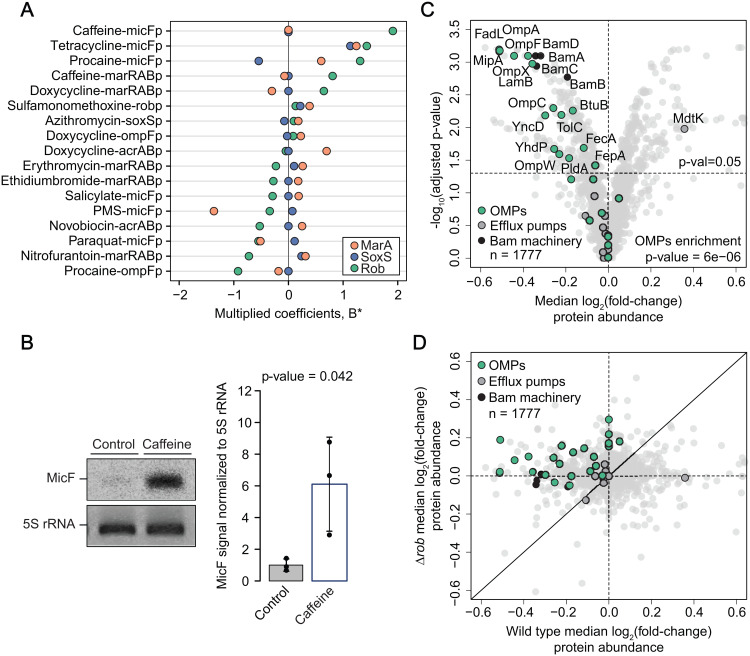
Caffeine induces proteome-wide changes in a Rob-dependent manner. (A) Caffeine-micFp interaction is primarily controlled by Rob. B* for all CPIs with non-zero Rob coefficients (n = 17), colored by regulator. (B) Caffeine increases MicF small RNA levels. Northern blot analysis confirming increased levels of small RNA MicF upon caffeine treatment (1 mM). One out of three biological replicates is shown. Quantification of the three replicates is shown as a barplot with the three replicates represented individually. Bar size and error bars reflect mean and standard deviation across the three replicates, respectively. P-value of a two-sided *t test* comparing the treated and untreated samples is shown. (C) Proteome-wide response to caffeine in *E. coli*. Volcano plot showing how -log_10_(p-value) relates to log_2_(fold-change) of caffeine treated compared to untreated cells. The p-values correspond to double-sided rank-sum test between fold-changes of a given protein and those of the entire set of proteins, after Benjamini-Hochberg correction for multiple testing. Median fold-changes across 6 caffeine concentrations at the two lower temperatures are shown (Materials and methods). Horizontal and vertical lines correspond to p-value = 0.05 and log_2_(fold-change) = 0, respectively. n refers to the total number of proteins detected. The double-sided Fisher’s exact test p-value for enrichment of OMPs among significantly decreased proteins is shown. (D) Comparison of proteome changes between wild type and Δ*rob* upon caffeine treatment. Horizontal and vertical lines correspond to log_2_(fold-change) = 0. The black line represents the 1-to-1 diagonal. n refers to the total number of proteins detected. Median across three replicates treated with increasing caffeine concentrations are shown (Materials and methods). The underlying data for all panels can be found in S7 Table.

To showcase the potential of our dataset for uncovering new molecular mechanisms, we chose to validate and further investigate the physiological consequences of caffeine-induced increase of MicF promoter activity – caffeine-micFp interaction (Fig 1E). Our choice was anchored to two points: first, it involves caffeine - a widely used food ingredient not previously known to impact transport regulation in prominent enterobacteria. Second, because caffeine-micFp interaction emerges from our dataset as the top CPI being primarily/exclusively controlled by Rob (Figs 3G and 4A), which is by far the least functionally characterized regulator, in comparison to MarA and SoxS. We started out by validating our initial observation that caffeine triggers *micF* expression. Indeed, MicF small RNA levels increase ~6-fold in the presence of caffeine when compared to no-caffeine control, as measured by northern blot (Fig 4B). Furthermore, our screen showed that caffeine-micFp interaction is lost in the absence of Rob, and we confirmed that *rob* complementation reverts this phenotype back to wild-type (S4A Fig). Our next step was towards understanding the mechanism by which caffeine triggers Rob regulatory activity. It has been previously suggested that Rob regulatory activity is triggered via post-translational modifications upon ligand interaction, either via direct interaction or foci dispersal [41,52,53]. Therefore, we first tested whether Rob could directly interact with caffeine in vitro using isothermal titration calorimetry (ITC), but no binding was detected (S4B Fig, Materials and methods). Next, we used in vivo thermal proteome profiling (TPP [54–57]), aiming to find caffeine-induced protein thermal stability changes that could explain the observed phenotype. In particular, we hypothesized that either direct binding or induction of Rob foci dispersal by caffeine, could cause Rob (de)stabilization in vivo. However, no substantial change in stability of Rob (or other proteins) was observed (S6 Table), precluding us to elucidate the mechanism of the caffeine-rob interaction at this stage. Nonetheless, our results show an extensive proteome adjustment in response to caffeine, with change in abundance of >200 proteins. Gene Ontology enrichment analysis (Materials and methods) revealed significant changes in few biological function GO categories (S4C Fig, Materials and methods), of which we found “protein insertion into membrane” of particular relevance. Specifically, the levels of all detected β-barrel assembly machinery proteins (Bam, 4 out of 5) are decreased upon caffeine treatment (Fig 4C). As the Bam machinery mediates the assembly of OMPs in Gram-negative bacteria, its decrease can potentially impact the levels of all OMPs. Indeed, we observed a strong enrichment of OMPs among all decreased proteins (Fig 4C), thereby suggesting that caffeine could strongly impact membrane permeability way beyond MicF and its targets. Finally, all protein changes upon caffeine treatment are lost in the absence of Rob (deletion mutant, Fig 4D), thereby ultimately establishing Rob as a key regulator of *E. coli* response to caffeine.

### Rob-dependent caffeine-micFp interaction underlies species-specific antibiotic antagonisms in *E. coli*

**Fig 5:**
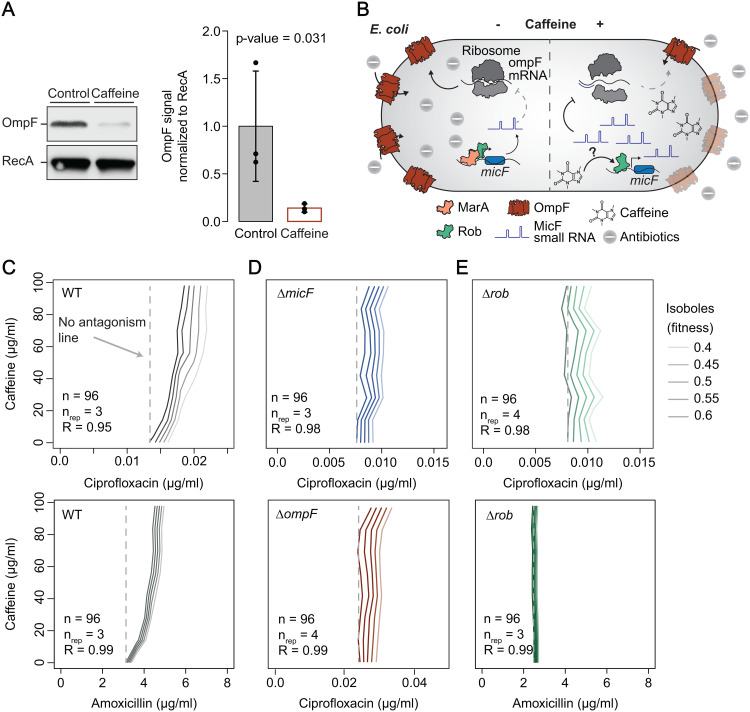
Rob-dependent caffeine-micFp interaction underlies species-specific antibiotic antagonisms in *E. coli.* (A) Caffeine decreases OmpF protein levels. Immunoblot analysis using whole cell lysate and an *E. coli* OmpF specific antibody shows OmpF decreased levels upon caffeine treatment (1 mM). One out of three biological replicates is shown. Quantification of the three replicates is shown as a barplot with the three replicates represented individually. Bar size and error bars reflect mean and standard deviation across the three replicates, respectively. P-value of a one-sided *t test* comparing the treated and untreated samples is shown. (B) Proposed model for the molecular mechanism of caffeine-ciprofloxacin antagonism. Caffeine triggers expression of MicF small RNA in a Rob-dependent manner, which then binds to the 5′-UTR of *ompF* mRNA to inhibit and decrease OmpF protein levels. This ultimately prevents ciprofloxacin from entering the cell, resulting in caffeine-ciprofloxacin antagonism. (C-E) Caffeine-antibiotic antagonisms in *E. coli* are *micF-*, *ompF*- and *rob*-dependent. Isobolograms for caffeine-antibiotic interactions for *E. coli* wild-type (C), Δ*micF* and Δ*ompF* (D) and Δ*rob* (E) are shown. Rightward oriented isoboles indicate antagonism, while upward oriented isoboles indicate no antagonism. A dashed line is plotted for no-antagonism reference for isobole 0.6 for all strains. One out of 3 or 4 biological replicates (n_rep_) is shown. R is the Pearson correlation between the biological replicates obtained with 96 (n) fitness values used to obtain each checkerboard. The underlying data for all panels can be found in S7 Table.

We next independently confirmed that the levels of one of the top decreased proteins upon caffeine treatment, OmpF, is indeed decreased in the presence of caffeine (Fig 5A). As OmpF is a major entry point for antibiotics in *E. coli*, we hypothesize that caffeine could decrease compound uptake (as we previously showed [13]) in a Rob-dependent manner, thereby causing antibiotic antagonism (Fig 5B). In order to test this hypothesis, we first re-evaluated the outcome of caffeine combination with ciprofloxacin and amoxicillin, two different antibiotics predominantly taken up through OmpF in *E. coli*, and which our previous work showed to be antagonistic [13]. Indeed, a checkerboard assay confirmed the antagonism, showing that the concentration of antibiotics needed to inflict a given inhibitory effect progressively increases with increasing caffeine concentrations (isoboles moving rightward, Fig 5C). For instance, amoxicillin IC_50_ (inhibitory concentration of 50%) increases by ~40% in the presence of 55.5 µg/ml of caffeine (S5A Fig), and caffeine alone has no inhibitory effect within the concentrations tested (S5B Fig). Next, we confirmed that deletion of *micF* or *ompF* mostly abolished the caffeine-ciprofloxacin antagonism (straight vertical isoboles, Fig 5D), confirming that these two molecular players are key to the antagonism. Furthermore, we also observed that caffeine antagonism with either ciprofloxacin or amoxicillin is strictly Rob-dependent, as *rob* deletion equally abolished both antagonisms (Fig 5E). Genetic complementation of *micF, ompF* and *rob* reverted the phenotypes close to wild-type again (S5C Fig). Importantly, deletion of *marA*, otherwise also upregulated by caffeine in a Rob-dependent manner, did not change the caffeine-ciprofloxacin antagonism (S5D–F Fig), further establishing Rob’s major role in regulating caffeine response in *E. coli*. Also here, marRABp induction by caffeine was re-established upon *rob* complementation (S5G Fig). In summary, our results suggest that caffeine is a novel and specific inducer of Rob transcriptional activity and this fully explains the molecular mechanism of caffeine-antibiotic antagonism in *E. coli*.

**Fig 6:**
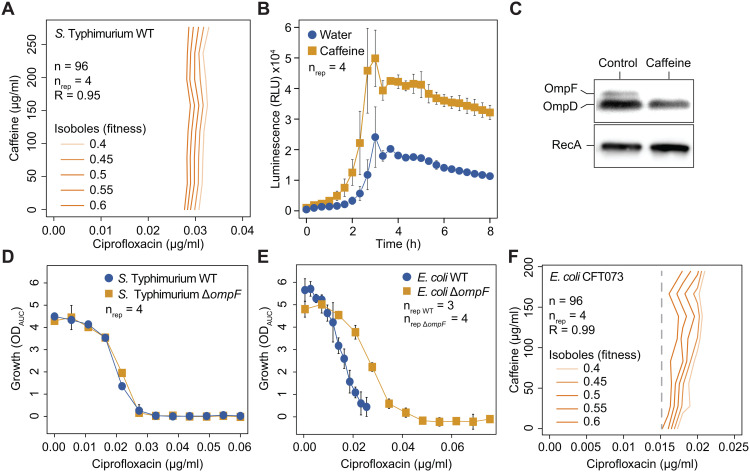
Caffeine-ciprofloxacin antagonism is absent in *S.* Typhimurium despite conserved regulatory mechanism. (A) Absence of caffeine-ciprofloxacin antagonism in *S.* Typhimurium. Isobologram for caffeine-ciprofloxacin for *S.* Typhimurium. Details as in Fig 4E. (B) Caffeine induces micFp activity in *S.* Typhimurium. Luminescence profiles of *S.* Typhimurium micFp reporter strain over time + /- caffeine (2 mM). Mean luminescence (dots) and standard deviations (error bars) over 4 biological replicates. (C) Immunoblot analysis using total protein extractions and an OmpF polyclonal antibody shows OmpF decreased levels upon caffeine treatment (2 mM). One out of 3 biological replicates is shown. (D) Ciprofloxacin MIC is not altered upon *ompF* deletion in *S.* Typhimurium. Ciprofloxacin MIC curves (growth vs. antibiotic concentration) of *S.* Typhimurium wild-type and Δ*ompF.* Mean growth (OD_AUC_, dots) and standard deviations (error bars) over 4 replicates. (E) Ciprofloxacin MIC increases upon *ompF* deletion in *E. coli*. Ciprofloxacin MIC curves (growth vs. antibiotic concentration) of *E. coli* wild-type and Δ*ompF.* Mean growth (OD_AUC_, dots) and standard deviations (error bars) over 3 or 4 biological replicates. (F) Caffeine-ciprofloxacin antagonism against a pathogenic *E. coli* strain. Isobologram for caffeine-ciprofloxacin for *E. coli* CFT073. Details as in Fig 5C. The underlying data for all panels can be found in S7 Table.

Interestingly, our previous work showed that antagonisms involving caffeine seem to be specific to *E. coli*, as they were not observed in the closely related species *Salmonella enterica* Typhimurium [13]. Here, too, we could confirm that caffeine does not change ciprofloxacin activity against *S.* Typhimurium (Fig 6A, as a control we also confirmed that caffeine alone does not inhibit the growth of *S.* Typhimurium S5B Fig). One possibility for this phenotype could be that, even though all regulators (MarA, SoxS, Rob and MicF) and effectors (MicF and OmpF) are also present in *S.* Typhimurium, their response to caffeine is different than in *E. coli*. Thus, we first assessed if the absence of caffeine antagonism in *S.* Typhimurium could be explained by lack of *micF* transcriptional induction by caffeine. We quickly disproved this hypothesis by using a reporter system to quantify the activity of the *micF* promoter (similar to the *E. coli*), as we observed that, also in *S.* Typhimurium, caffeine treatment increases micFp expression (Fig 6B). In addition, we could also confirm that caffeine treatment reduces the level of OmpF in *S.* Typhimurium (Fig 6C), but not necessarily of other OMPs, as it occurs in *E. coli*. It has been previously suggested that OmpF-mediated uptake is not as determinant for ciprofloxacin activity against *S.* Typhimurium as it is for *E. coli* [58]. Indeed, we observed that deletion of *ompF* alone (no addition of caffeine) decreases ciprofloxacin sensitivity in *E. coli* but not in *S.* Typhimurium (Fig 6D, E), suggesting that lack of conservation of antagonism with caffeine is likely due to different antibiotic uptake mechanisms between the two species. Altogether, this example illustrates how challenging it is to predict final outcome of CPIs on drug transport across species, even closely related ones, for which the regulatory response to the environmental cues is conserved. Nonetheless, our previous work showed that ciprofloxacin antagonism with caffeine occurs also for a gut commensal *E. coli* strain [13], suggesting that the mechanism could be more general within *E. coli*. While not extensively assessing this question, we could confirm that the antagonism occurs also in a urinary tract infection *E. coli* isolate (Fig 6F), suggesting that it could be relevant for commensal and pathogenic *E. coli* across different niches in the human host.

## Discussion

Transport across the bacterial cell envelope has been subject of study for decades, with important constituents like AcrAB-TolC and OmpF, and also central regulators such as MarA, SoxS and Rob being extensively characterized [2,15]. Despite vast molecular evidence on how each of these proteins play a role in a complex regulatory cascade, we mostly lack comprehensive approaches that provide a general overview on how all the different players come together to orchestrate a coordinated response. Here we provide an integrative systematic approach to assess the transcriptional response of a small set of transport-related genes to environmental cues (CPIs), while quantitatively assessing regulator contributions to each response. Such an approach enabled not only the discovery of new CPIs, such as caffeine-micFp and macrolides-marRABp, but most importantly, allowed us to disentangle the relative contribution of each central regulator to the observed phenotype. Our results endow several previous observations, such as that not all compounds being effectively effluxed [2,3,59] are capable of inducing expression of efflux genes, or that transport regulation underlies antibiotic antagonism [13]. Importantly, we provide a set of new general findings suggesting a paradigm change in our perception of how *E. coli* controls its transport. For instance, *marA* regulation is mostly investigated in the presence of salicylate, which releases *marA* transcriptional repression by binding MarR [26]. However, other compounds seem to regulate *marA* in a growth dependent manner, even in the absence of MarR. Another interesting finding is that ~1/3 of all CPIs depend on Rob. This is well beyond what is generally described or perceived, and indicates that a much better understanding of Rob-dependent regulation is crucial to fully comprehend transport transcriptional control. Interestingly, almost all CPIs involving Rob also involve MarA, or MarA and SoxS (Fig 3E), which certainly renders full functional characterization more challenging. Exceptionally, our data suggests that Rob alone seems to have complete control of caffeine response in *E. coli* way beyond transcription, where we observed significant changes in the abundance >200 proteins, including several OMPs, in a Rob-dependent manner. Despite several attempts, we were not yet able to decipher the molecular mechanism by which caffeine triggers Rob transcriptional activity and its downstream effects. Similarly, at this stage it is still unclear how exactly caffeine induces such a proteome-wide adjustment via a single regulator. Beyond caffeine and Rob, we readily acknowledge that other regulators than those we selected will most certainly also play a role in controlling the transcriptional response of the promoters we examined, and also that the compounds we used will most certainly inflict wider transcriptional changes than those we monitored. Nonetheless, this study, even if limited to 7 promoters, shows the potential of our approach to generate valuable insight into the extensive cross-talk of complex transcriptional regulatory networks.

A second aspect to highlight is the potential of our dataset for mining molecular mechanisms. We showcased it by exposing how caffeine-micFp interaction underlies caffeine-antibiotic antagonism through impaired antibiotic uptake, in a Rob-dependent manner. Even if caffeine causes only a modest increase in antibiotic resistance in vitro (up to~40%), this finding features the potential impact of the immediate environment on treatment efficacy through transport modulation, and further investigation is needed to assess clinical consequences. Given the ever increasing evidence of transport’s prevalent role in the bacterial response to harsh environments, for instance in modulating the impact of human-targeted-drugs in gut microbes [10], or potentially contributing to bioaccumulation within the gut microbiota thereby modulating drug action on the host [11], the scope of our findings and approach goes well beyond *E. coli* or even enterobacteria. Nonetheless, our data indicate that the ultimate physiological consequences of (even) conserved compound-promoter interactions are not necessarily conserved across species. More specifically, we showed that the caffeine-micFp interaction and the subsequent decrease in OmpF levels are conserved between *S.* Typhimurium and *E. coli*, but this is not sufficient to yield caffeine antagonism in *S.* Typhimurium. Several reasons could explain this phenomenon, for instance the presence and alternative regulation by caffeine of additional outer membrane porins in *S.* Typhimurium, or a more prevalent role of efflux versus uptake [58,60,61]. Based on these findings, we foresee a challenging, but unavoidable and important task in mapping key determinants of transport functions across different bacteria. Nonetheless, our data supports that caffeine antagonisms extend to pathogenic *E. coli* strains, advocating for further investigation of its potential relevance in host contexts.

## Materials and methods

### Growth medium, reporter plasmids and strain construction

A summary of all strains used in this study can be found in S1 Table. *Escherichia coli* BW25113 and *Salmonella enterica* subsp. *enterica* ser. Typhimurium 14028S were used as wild-type strains (WT) and cultured in Lysogeny Broth (LB Lennox) adjusted to pH 7.5 at 37°C. The medium was supplemented with kanamycin (50 µg/ml, CatNo. K1876-5G, Sigma-Aldrich-Merck), carbenicillin (100 µg/ml, CatNo. Cay20871−5, Biomol) or spectinomycin (100 µg/ml, CatNo. S4014-5G, Sigma-Aldrich-Merck) when selection was required for strain construction.

All plasmids constructed in this study are listed in S1 Table. Promoter regions (500 bp upstream of respective start-codon) of interest were amplified from genomic DNA of *E. coli* BW25113 or *Salmonella* Typhimurium using Q5 polymerase according to supplier instructions (CatNo. M0491S, New England Biolabs (NEB), USA). All DNA oligos used in this study are listed in S1 Table. Reporter plasmids for *E. coli* were assembled by restriction-ligation using enzymes EcoRI and XhoI (SalI in case of acrABp, CatNos. R3101S, R0146S, R3138S, NEB) to linearize the backbone vector pTU175-LUX. T4 DNA ligase (CatNo. M0202S NEB) was used for plasmid assembly following supplier instructions. Reporter plasmids for *S.* Typhimurium were assembled using Gibson Assembly, using XhoI for plasmid linearization. Vector pTU175-LUX is a low copy plasmid with a pSC101 origin of replication constructed from pTU175 [62]. by insertion of an oriT, a spectinomycin resistance cassette and the full luxCDABE operon (amplified from Plasmid #44,918, AddGene) with a putative RBS for basal expression – used as **e**mpty **v**ector **c**ontrol, EVC. The pASCOT-LUX vector is a variant of pTU175-LUX with a carbenicillin resistance cassette instead of spectinomycin used for *Salmonella*. DNA inserts were digested with the respective restriction enzymes and assembled into the plasmid backbone utilizing T4 DNA ligase according to a standard protocol (NEB). Plasmids were transformed into *E. coli* BW25113 and *Salmonella* Typhimurium 14028S by transformation (TSS transformation) and electroporation, respectively. The protein expression vector for purification of Rob-6xHis was created by insertion of a PCR fragment containing *rob* into pET28a using restriction-ligation as mentioned above with Hind-III and NcoI (CatNos. R3104S, R3193S NEB). The plasmid was subsequently transformed into *E. coli* BL21 (DE3).

Deletions of ∆*marA*, ∆*soxS*, ∆*micF* and ∆*ompF* were derived from the KEIO collection in case of *E. coli* [63] or a similar knockout-library in case of *S.* Typhimurium [64]. Mutations were confirmed using PCR and transduced into wild type BW25113 or ST14028s using P1 and P22 phage, respectively. Deletion of *rob* in *E. coli* was done with Lambda RED recombineering according to the protocol of Datsenko and Wanner [65], similar to how the KEIO collection strains were created. Kanamycin resistance cassettes were subsequently removed using the pCP20 plasmid.

Genetic complementation of ∆*rob* and ∆*ompF* was achieved by assembling PCR products of wild type *rob* and *ompF* with 500 bp of respective promoter regions into the pKD13 backbone using Gibson Assembly [66]. PCR fragments containing respective genes, kanamycin cassette and FRT sites were then transferred to the native locus of respective deletion strains using Lambda RED recombineering [65]. Positive clones were selected using kanamycin as a selection marker and gene insertions were subsequently transferred into clean backgrounds using P1 transduction, followed by excision of the kanamycin cassette using plasmid pCP20. Deletion of *micF* was complemented using a version of the pTU175 vector, where the *luxCDABE* operon was replaced by *micF* with its respective 500 bp promoter region. The resulting pTU175-*micF*-comp was transformed into the Δ*micF* deletion background using TSS transformation [67]. All strains and plasmids constructed during this study are available from the authors upon request.

### Compound-promoter screens

The compound-promoter screen for *E. coli* BW25113 (wild type, as well as Δ*marA*, Δ*soxS* and Δ*rob* backgrounds) containing each reporter plasmid – arcABp, marRABp, soxSp, robp, micFp, ompFp, tolCp and EVC - was done in black 384 well-plates with clear bottom (CatNo. 781097, Greiner-Bio One, Germany), in 40 µl LB Lennox. The compound library contains 94 diverse compounds purchased from Biomol (Germany), MP Biomedicals (Germany), or Sigma-Aldrich-Merck (solvents, concentrations and purchase details listed in S2 Table). The highest screening concentration of compounds with antimicrobial activity was adjusted to MIC for antimicrobials, 500 µM for most non-antimicrobials, and up to 1 mM for small compounds with similarity to canonical inducers (positive controls, e.g., salicylate, S2 Table). Four working concentrations were achieved through 1:2 serial dilutions using a Biomek i7 liquid handler (Beckman Coulter, S1A Fig). Precultures were grown overnight and diluted to an OD_600 nm_ of 1 (WT) or 0.1 (deletion backgrounds) and used to inoculate 384 well-plates using a Singer Rotor+ replicator (Singer Instruments, UK), resulting in further ~1:600 dilution and starting OD of ~0.003 and ~0.0003. Transparent breathable membranes (Breathe-Easy, Sigma-Aldrich-Merck) were used to seal plates, which were then incubated at 37°C, shaking at 800 rpm using a Cytomat 2 incubator (Thermo Scientific). Growth (OD_600 nm_) and reporter activity (luminescence) were measured in 30 minutes intervals over 12 hours in a Synergy H1 plate reader (Agilent, USA). The screen was performed in biological duplicates, resulting in 768 growth and luminescence curves per strain. The entire dataset (raw data) is provided in S1 and S2 Data.

Data analysis was performed using R (version 4.2.2). A representation of the analysis pipeline can be found in S1B Fig. Baseline-correction of growth curves was done by subtraction of initial OD_600 nm_ before growth onset (time 1–2 hours). Area under the curve was calculated for growth (OD_AUC_) and luminescence (Lux_AUC_) curves between 0 and 8 h in case of the wild type and between 1 and 9 h for the deletion background strains to account for the differences in inoculum size mentioned above. This eight-hour interval covers lag phase, exponential phase and transition into stationary phase assuming regular, non-stressed growth – using water instead of any compound. Non-growing samples (less than 10% of median OD_AUC_ across all compounds, reporters and concentrations) were removed from further analysis (1,467 wells out of 24,576 wells in total (~6%), listed in S3 Table), and luminescence was then normalized by growth (Lux_AUC_/OD_AUC_, S3 Table). Importantly, no compound was completely exlcuded. Based on the premise that most compounds in the library do not induce/inhibit expression of any of the promoters, compound-promoter interactions were defined to be the deviation (residuals) of the line-of-best fit (Huber robust linear regression) [68] of normalized luminescence between a given promoter and the EVC. This method allows to better control for possible non-specific transcriptional effects of each compound, as we observed that normalized luminescence for EVC can in fact change across compounds/concentrations (S3 Table). Importantly, we observed that the robust linear fits had reasonably high R^2^ (coefficient of determination, S4 Table), indicating that this approach captures and corrects well non-specific effects, and positive and negative deviations (residuals) reflect compounds which increase or decrease promoter expression, respectively. The interaction scores (residuals) were subsequently Z-transformed (Z-scores) to allow comparability of promoters of varying signal intensity. Finally, significant compound-promoter interactions were called based a double cut-off on mean Z-score of all compound concentrations and replicates for each compound-promoter pair (±1) and Benjamini-Hochberg [69] adjusted double sided rank-sum test p-value (<0.05) comparing the Z-scores distributions of each compound-promoter to water-promoter. Most mean Z-scores are calculated with all 8 values (4 concentrations x 2 replicates), while in some cases data of up to three concentrations was removed due to poor growth as detailed earlier. However, phleomycin is the only case calculated with only 2 values (3 out of 4 concentrations over-killed) and there is no case where a compound was completely removed in the wild-type background. In all deletion backgrounds phleomycin became lethal at all four concentrations and was removed entirely as the only drug. All removed wells are indicated in S3 Table.

### DNA and RNA quantification by qPCR and RT-qPCR

To confirm plasmid copy number stability after treatment with protein biosynthesis inhibitors, overnight cultures of *E. coli* BW25113 harboring plasmids pTU175-Lux-EVC or pBR322 were diluted 1:100 in fresh LB medium and grown at 37°C with agitation until exponential phase (OD _600 nm_ ~ 0.4). Chloramphenicol was added to half of the cultures to a final concentration of 2 µg/ml followed by further cultivation for 30 min. Total DNA was extracted using the Monarch Genomic DNA Purification Kit (NEB) using the manufacturer’s instructions. All experiments were conducted in three biological replicates. Relative plasmid number fold changes were estimated by comparison with a non-treated control. All DNA oligos used in this study are listed in S1 Table.

To confirm induction of *marA* after treatment with salicylate clarithromycin and sulfamethoxazole, overnight cultures of wild-type *E. coli* BW25113 were diluted 1:100 in fresh LB medium and grown at 37°C with agitation until exponential phase (OD _600 nm_ ~ 0.4). Salicylate, clarithromycin and sulfamethoxazole were added to cultures to a final concentration of 1 mM, 40 µM and 0.8 mM, respectively, while controls were left untreated, followed by further cultivation for 30 min. Total RNA was extracted using the Monarch Total RNA Miniprep Kit (NEB) using the manufacturer’s instructions. Luna Universal One-Step RT-qPCR Kit (NEB) was used to prepare cDNA and as reagent for RT-qPCR according to the manufacturer’s instructions. All experiments were conducted in at least three biological replicates and relative expression levels were estimated as previously described [70], using *gyrA* expression as reference.

### Lasso regression for estimation of regulator contributions of compound-promoter interactions

Firstly, the interaction scores gathered from the different genetic backgrounds were quantile normalized to account for baseline changes in expression due to regulator deletions. These normalized scores were then further pre-processed to consider the baseline values obtained from exposure to water through a soft-thresholding approach. Namely, we subtract the value of the water-promoter score from compound-promoter scores gathered in the same genetic background. In cases where the compound-promoter score is lower than the corresponding water-promoter score, the compound-promoter score is set to 0. These normalized and water-thresholded scores are then centered and scaled prior to modelling.

Once pre-processed, we modelled the scores for a given CPI as a function of compound concentration and genetic background, resulting in a design matrix X= [$X_{conc},X_{rob},X_{marA},X_{soxS}$] with dimensions nx4 (n being the number of samples). Compound concentration is a discrete variable ($X_{conc}\in \{1,2,4,8{\}}^{n}$), while genetic background is represented as a binary variable to indicate regulator presence/absence ($X_{rob,}X_{marA},\;X_{soxS}\in \{0,1{\}}^{n}$). We also include all pairwise interactions between the variables in X in our model. In this way, our model can be stated as:


$$Y=\beta_{0}+\sum_{j}^{4}\beta_{j}X_{j}+\frac{1}{2}\sum_{j\neq k}^{4}\theta_{jk}X_{j}X_{k}+\varepsilon$$

where $Y\in {\mathbb{R}}^{n}$ is the vector of pre-processed interaction scores for a given CPI, $\beta_{0}$ is a CPI-specific intercept$,\beta_{j}$ is the effect that variable j∈{conc,rob,marA,soxS} has on Y, $\theta_{jk}$ captures the pairwise interaction effect between variables j and k, where j,k∈{conc,rob,marA,soxS} and j≠k, and ε models technical and biological noise.

To select for a parsimonious model, we estimate model coefficients via regularized maximum-likelihood estimation with lasso penalization [48,49]. Furthermore, we restrict non-zero interaction terms $\theta_{jk}$ to only be present if both associated individual effects $\beta_{j}$ and $\beta_{k}$ are also non-zero (strong hierarchy). This restriction prioritizes explaining Y in terms of main effects β, and interactions are only included if the response cannot be solely captured by linear additive effects.

Given that the log-likelihood of our model is $l(\beta_{0},\beta ,\theta )=||Y-\beta_{0}-X\beta -\frac{1}{2}X\theta X^{T}{\|}_{2}^{2}$, the complete optimization problem for the hierarchical interaction model is:


$$min_{\beta ,\theta }l\left(\beta_{0},\beta ,\theta \right)+\lambda ||\beta |{|}_{1}+\frac{\;\lambda }{2}||\theta |{|}_{1}$$

$$s.t.\theta =\theta^{T},||\theta_{j}|{|}_{1}\leq \left|\beta_{j}\right|$$

where λ>0 is the lasso penalization parameter that controls the sparsity of coefficients β and θ.

We solve this optimization problem using the efficient implementation provided in the R package hierNet (version 1.9) [48]. The optimal value for λ was determined through 4-fold cross validation. We selected the λ value that was within one standard error of the λ that minimized the cross validation error. In this way, the nature of the individual effects that drug concentration and regulator presence have on changes in gene expression in response to a given chemical stressor are captured in the sign and magnitude of the coefficients β.

Interaction coefficients θ, potentially reflecting added synergistic effects, were negligible compared to concentration or single regulator coefficients, and were therefore excluded from further analysis. Regulator contribution coefficients (β) to compound-promoter pairs were then multiplied by the absolute of mean Z-scores of the wild type – multiplied coefficients B*– thereby reflecting the strength of the compound-promoter interactions in wild type, in addition to regulator contribution. This approach facilitates interpretation and enables better understanding of regulator contributions to strong compound-promoter interactions in the wild type.

We performed a 10-fold cross-validation to provide a robustness measurement of each compound-promoter pair’s regulator contributions using a “out-of-sample R^2^” metric. In this procedure, the data for each compound-promoter pair was randomly divided into a training set (75% of the data) and a testing set (25% of the data). The model was trained on the training set, and its out-of-sample performance was evaluated on the testing set. The average R^2^ per compound-promoter pair was estimated considering the results from folds where the ground-truth test set values had a variance > 0 (i.e., more than one unique value). For compound-promoter pairs for which all contribution coefficients are invariably zero, R^2^ cannot be estimated.

A summary of the relevant data from the statistical model is provided in S5 Table.

### Determination of Minimum Inhibitory Concentration (MIC)

Compound Minimum Inhibitory Concentration (MIC) against *E. coli* and *Salmonella* strains was quantified using liquid growth assays in LB-Lennox in microtiter plates in preparation for screening or checkerboard assays. The assay was performed in 96 well-plates in 100 µl, and conditions similar to those used in the screening approach described above. Drugs were diluted linearly in 11 equal steps, allowing for finely resolved quantification of antimicrobial efficacy. Growth curves were analysed similar to those from the screening approach, and growth after 8 h was approximated by OD_AUC_.

MIC curves were generated to assess complementation of *micF*, *ompF* and *rob* in the respective deletion genetic backgrounds. We used our checkerboard assays to select a suitable caffeine concentration where antagonism was observed for the wild type: at 55.5 µg/ml caffeine the ciprofloxacin and amoxicillin MIC curves are shifted towards higher concentrations (S5A Fig). We then followed the procedure described above (in 384 well-plates, 40 µl) for obtaining MIC curves for ciprofloxacin and amoxicillin with the complemented mutants in the presence and absence of 55.5 µg/ml caffeine. The MIC curves were estimated as previously described [71] by fitting a three-parameter logistic model to the drug dosage response curves using the R-package drc [72], using OD_AUC,8h_ as proxy for growth. Raw data for the MIC curves is provided in S4 Data.

### Isothermal Titration Calorimetry

Purification and subsequent ITC of Rob-6xHis with caffeine were carried out essentially as described previously [41]. Briefly, Rob was expressed in *E. coli* BL21 (DE3) from a pET28a plasmid harboring the *E.* coli Rob open reading frame, fused to a C-terminal 6xHis-tag. 4x 2 l cultures in LB medium were grown at 37°C to an OD_600_ of 0.6 and expression was induced by addition of 1 mM isopropyl β-D-1-thiogalactopyranoside (IPTG), followed by protein expression at 18°C overnight. Pellets were harvested by centrifugation and resuspended in lysis buffer (50 mM Tris-HCl pH 8.0, 200 mM NaCl, 10 mM imidazole, 1 mM TCEP, 10% glycerol, protease inhibitors, DNase), followed by lysis via sonication and centrifugation at 4°C at 30,000 x *g* for 30 min. The soluble lysate was then loaded onto 5 ml Ni-NTA agarose beads. After intensive washing of the beads, the Rob protein was eluted with IMAC elution buffer (20 mM Tris-HCl pH 8.0, 200 mM NaCl, 250 mM imidazole, 1 mM TCEP, 10% glycerol). As a second purification step, target protein containing IMAC eluates were pooled and subjected to size-exclusion chromatography on a HiLoad Superdex 75 16/600 pg column, equilibrated to SEC buffer (200 mM NaCl, 20 mM Tris-HCl pH 8.0, 10% glycerol, 1 mM DTT). SEC peak eluates were pooled and concentrated to 14.35 mg/ml. Samples were aliquoted, frozen in liquid nitrogen and stored at −80°C.

Isothermal titration calorimetry (ITC) experiments were done using a MicroCal PEAQ-ITC titration calorimeter (Malvern) equilibrated to 25°C. Rob was adjusted to 50 μM in dialysis buffer (20 mM HEPES, 200 mM NaCl, pH 8). Caffeine solutions were prepared fresh in the same buffer to a final concentration of 10 mM. The experimental parameters used with the PEAQ-ITC system were 18 times 2 μl injections at 4 seconds duration, 150 seconds spacing, 750 rpm stirring speed, and a reference power of 10 μcal/s.

### Proteomics

In vivo two-dimensional thermal proteome profiling (2D-TPP) with wild type *E. coli* BW25113 and Δ*marA* backgrounds was performed as previously described in [55,56], with a caffeine concentration gradient ranging from 0 - 400 µM (0, 4.1,10.2, 25.6, 64, 160, 400 µM). A single culture of mid-exponential cells was split before treatment. Protein digestion, peptide labelling, MS-based proteomics and data analysis for assessment of protein thermal stability changes were performed as previously described [55–57]. Caffeine-induced changes in protein abundance without temperature denaturation were calculated as the median fold-change across all caffeine concentrations (4.1 - 400 µM) at the lowest two temperatures (42°C, 45.4°C, n = 12). Benjamini-Hochberg adjusted double-sided rank-sum test was used to compare the fold-change distributions of each protein to the background (entire dataset).

Gene Ontology (GO) analysis (for biological process) was performed to identify functional enrichment among significantly abundant proteins (p-value < 0.05) in *E. coli*. Gene annotations were obtained from EcoCyc (https://ecocyc.org), and are provided in S6 Table. Statistical enrichment analysis among all detected proteins (n = 1,177) was performed using Fisher’s exact test. No correction for multiple testing was done, as the p-values were generally high (low risk for false positives), and a correction would deem all changes non-significant, thereby increasing the risk for false negatives. Thus, GO terms with a p-value < 0.05 were considered significantly enriched.

### Quantification of OmpF levels by Western blot

*E. coli* and *S. enterica* OmpF levels were quantified by Western blotting. Caffeine treated and untreated cultures were prepared in three biological replicates each by 1:100 dilution of over-night cultures in 20 mL LB-medium and grown at 37°C and 180 rpm shaking. Caffeine was added to half of the flasks at a concentration of 1 mM, while the other half of the flasks served as a negative control. Cultures were grown until OD_600_ of ~0.8, followed by pelleting via centrifugation (10 min, 4,000 x g, 4°C). Pellets were resuspended in 200 µl 4% SDS solution, heated to 95°C for 5 min and stored at −80°C until used. The Pierce BCA Protein Assay Kit (Fisher Scientific, Germany) was used to calculate total protein concentration of all samples according to the manufacturer’s instructions. Samples were adjusted to 4 µg/µl protein with 4% SDS solution and mixed in equal parts with 2x Laemmli buffer containing 2-mercaptoethanol. Aliquots containing 20 µg of total protein (10 µl) were boiled for 5 min at 95°C to denature any proteins. Samples were separated by SDS-PAGE on a 10% gel at 80 V for 3.5 h and transferred to a PVDF membrane via Trans-Blot Turbo system (BioRad), according to the manufacturer’s instructions. A specific rabbit αOmpF antibody (kindly provided by Trevor Lithgow, Monash University, Australia) and a rabbit αRecA antibody (ab63797, Abcam) were used as primary antibodies at dilutions of 1:20,000 and 1:5,000, respectively, to detect OmpF and RecA on the PVDF membrane. For both primary antibodies, we used an HRP-coupled secondary antibody (A0545, Sigma), and Pierce ECL Western Blotting-Substrate (Thermo Scientific) to visualise OmpF using an chemiluminescence imager (Intas Science Imaging Instruments GmbH, Germany).

### Quantification of MicF small RNA by Northern blotting

Bacterial cultures were grown to exponential phase (OD_600_ ~ 0.4) and treated with 1 mM caffeine for 30 min or left untreated as negative control. Samples were then mixed with 0.2 volumes of STOP solution (95% ethanol, 5% phenol) and snap-frozen in liquid nitrogen to prevent RNA degradation. Total RNA was extracted using the hot phenol method. Cell pellets were thawed and resuspended in 65°C lysis buffer (40 mM EDTA pH 8, 200 mM NaCl, 0.5% SDS, 100 mM Tris-HCl pH 7.5), incubated for 5 min at 65°C in a water bath and subsequently mixed with acidic phenol (ROTI Aqua-Phenol, Roth). Samples were mixed thoroughly by vortexing, snap-frozen in liquid nitrogen and centrifuged for 10 min. The upper aqueous phase was then mixed with the same volume of chloroform-isoamyl alcohol (24:1) and mixed again by vortexing. The resulting upper phase was then mixed with 1/10th volume of 3M sodium acetate (pH 4.5) and one volume isopropanol to precipitate total RNA for 30 min on ice. Supernatants were subsequently removed, pellets dried and resuspended in RNAse free water. Northern blotting, radioactive labelling of DNA oligonucleotides, hybridization and signal detection were all performed as previously described [73]. Signals were subsequently analysed using ImageJ software [74,75].

### Checkerboard assays

Quantification of interactions between caffeine and ciprofloxacin and/or amoxicillin was performed using checkerboard assays. In brief, a checkerboard assay resembles a two-dimensional MIC assay, with two different drugs being combined across concentration gradients. The assays were performed in 96 or 384 well-plates in biological quadruplicates and conditions similar to the screening described above. Growth inhibitory effect (OD_AUC_ after 8h) was determined in a series of 7 (vertical dilution series) equally spaced concentrations for caffeine and 11 (horizontal dilution series) equally spaced concentrations for the antibiotic. Concentrations were adapted for *E. coli*, *S. enterica*, and respective deletion mutants (*Δrob*, Δ*micF*, Δ*marA* and Δ*ompF*). Fitness was calculated by normalization of OD_AUC_ of each well with the no-drug control. Lines of equal fitness (isoboles) were estimated by the contours derived from drug-interaction-surfaces. Provided that caffeine alone does not show inhibitory effects at the concentrations tested, antagonism with ciprofloxacin or amoxicillin is reflected by increased concentrations of ciprofloxacin or amoxicillin needed to inflict a given inhibitory effect with increasing concentrations of caffeine (isoboles moving rightward, Fig 4E). Raw data for all checkerboards is provided in S3 Data.

## Supporting information

S1 Fig Schematic representation of the screen workflow and data processing. (A) Schematic screen workflow. Details described in Materials and methods. (A) Schematic of data processing. Details described in Materials and methods. (C) Boxplots of growth (OD_AUC_) across all reporters and replicates. Each boxplot represents a 384 well-plate (n = 384). Negative controls (water treatment) are displayed in black (n = 8 per strain). 1 and 2 refer to biological replicates. Center, upper and bottom lines represent 25^th^, 50^th^ and 75^th^ percentiles, whiskers extend to 1.5x inter-quartile range (IQR) and points beyond whiskers are represented individually. (D) Boxplots of luminescence (AUC_LUX_) data across all reporters and replicates. Each boxplot represents a 384 well-plate (n = 384). Negative controls (water treatment) are displayed in black (n = 8 per strain). 1 and 2 refer to biological replicates. Center, upper and bottom lines represent 25^th^, 50^th^ and 75^th^ percentiles, whiskers extend to 1.5x IQR and points beyond whiskers are represented individually. (E) Treatment with protein biosynthesis inhibitors does not affect copy number of pTU175 plasmids. Relative fold-change of pTU175-Lux-EVC and pBR322 after treatment with 2 µg/ml chloramphenicol compared to a negative control using qPCR. Three biological replicates are shown, and the line represents the mean. (F) Pearson replicate correlation of growth (OD_AUC_), luminescence (LUX_AUC_) and normalized luminescence (LUX_AUC_/OD_AUC_) between the duplicates of each strain. Line represents the mean replicate correlations for each variable. The underlying data for all panels can be found in S7 Table.
(PDF)

S2 Fig General principles driving CPIs in *E. coli*: additional supporting findings. (A) General features of CPIs: number of CPIs per compound classified as up and down-regulation. (B) Previously known CPIs paraquat-soxSp [17] and procaine-micFp [31] are captured by our screening approach. Growth (OD_600 nm_) and luminescence (RLU) profiles over time for soxSp (top) and micFp (bottom) basal activity (grey) and with increasing concentrations of paraquat or procaine, respectively (conc, S2 Table) are shown. Mean values of two biological replicates are shown. (C) Clarithromycin and sulfamethoxazole are novel inducers of marRABp expression. RNA levels of *marA* after treatment with salicylate (positive control), clarithromycin and sulfamethoxazole. Data was double normalized to a non-treated control and to the house-keeping gene *gyrA* (Materials and methods). Three biological replicates are shown, and the line represents the mean. (D) Correlation between growth and promoter activity for EVC, acrABp and robp. Z-scores of all compound-EVC/acrABp/robp tested pairs including water across all 4 concentrations and 2 biological replicates (n) are plotted against growth (OD_AUC_). Pearson correlation coefficients (R) indicate no-, negative and positive correlation for EVC, acrABp and robp, respectively. Correlation p-value (double sided *t test*) shown. Linear relationships are illustrated by lines of best fit (Huber robust model). (E) Chemical structures of known and novel marRABp inducing compounds. (F) Induction of marRABp by sulfamethoxazole, as well as its negative correlation with growth, are independent of MarR. Luminescence profiles over growth were measured across a linear range of sulfamethoxazole concentrations from 0 µg/ml to 101.2 µg/ml in wild-type and ∆*marR*. Growth-normalized luminescence is plotted against growth for two independent biological replicates, and lines-of-best-fit (pooled replicates) are shown to highlight strong correlation between the two variables. The underlying data for all panels can be found in S7 Table.
(PDF)

S3 Fig Assessing contributions of MarA, SoxS and Rob to compound-promoter interactions. (A) Deletion of *marA*, *soxS* or *rob* sensitizes bacteria to several compounds at sub-inhibitory concentrations. Top: boxplots of the residuals of the lines-of-best-fit between growth of the regulator mutants and wild-type for all tested compound-promoter pairs including water across all 4 concentrations and 2 biological replicates (median normalized OD_AUC_ within each strain). Only data from the promoterless control (EVC) is used. Negative residuals represent compound concentrations to which the regulator mutant is more sensitive than the wild-type. White boxes represent all pairs, and grey boxes correspond to the subset of pairs with 0.2 < wild-type median normalized OD_AUC_ < 0.7, respectively. The number of data points is indicated below each box plot. Boxplots indicate 25^th^, 50^th^ and 75^th^ percentiles, and whiskers extend up to 1.5x the interquartile range (IQR) from the 25^th^ and 75^th^ percentiles. p-value from a one-sided statistical *t test* comparing full and subset residuals per mutant are shown. Bottom: Residuals of the lines-of-best-fit between growth (median normalized OD_AUC_) of Δ*marA* and wild-type for all tested compound-promoter pairs across all 4 concentrations, including water and 2 biological replicates plotted against growth of the wild-type (median normalized OD_AUC_). Only data from the promoterless control (EVC) is used. Grey region corresponds to 0.2 < wild-type median normalized OD_AUC_ < 0.7. **(B)** Schematic of the Lasso regression model to estimate regulator contributions to CPIs. Details described in Materials and methods. **(C)** Boxplots of contribution coefficients b and q grouped by name. Center, upper and bottom lines represent 25^th^, 50^th^ and 75^th^ percentiles, whiskers extend to 1.5x IQR and points beyond whiskers are represented individually. Due to the nature of the data – very sharply zero-centered – 25^th^, 50^th^ and 75th overlap. **(D)** Scatterplot of out-of-sample R^2^ versus wild-type mean Z-scores for all compound-promoter pairs. CPIs are represented with darker color. **(E)** CPIs have higher than background out-of-sample R^2^. Boxplot of out-of-sample R^2^ for all compound-promoter pairs and for CPIs. Center, upper and bottom lines represent 25^th^, 50^th^ and 75^th^ percentiles, whiskers extend to 1.5x IQR and points beyond whiskers are represented individually. **(F)** Scatterplot of multiplied coefficients B* versus model coefficients b of single regulator contributions, colored by regulator. **(G)** Correlation of acrABp promoter activity with growth is lost upon *marA* deletion. Pearson correlation coefficients of Z-scores versus growth (OD_AUC_) for each individual promoter in Δ*marA*. Correlation p-value (double sided *t test*) shown above bars. **(H)** acrABp and marRABp promoter activities are strongly correlated in a MarA-dependent manner. The mean Z-scores of all pairs including water (n = 384) of acrABp plotted against marRABp. Correlation p-values (double sided *t test*) and Pearson correlation coefficien*t*s are shown. Inlay shows b_*marA*_ to acrABp promoter activity in the wild-type for all CPIs involving acrABp. The underlying data for all panels can be found in S7 Table.
(PDF)

S4 Fig Supporting material for rob-caffeine interaction. (A) Complementation of *rob* in the Δ*rob* mutant re-enabled activation of micFp transcriptional activity upon caffeine treatment (as measured by our luminescence reporter), as in the wild-type. Luminescence (RLU) profile over time for micFp basal activity (water) and upon treatment with caffeine in 96 well-plates are shown. Average across 3 biological replicates is shown, error bars represent standard deviation (albeit very small, and therefore not visible). (B) Caffeine does not interact with Rob. The plot shows a binding isotherm representing the integrated heats (after baseline and dilution correction) over increasing molar ratio of caffeine-Rob, as obtained by ITC. (C) Gene Ontology enrichment analysis for protein abundance changes upon caffeine treatment in *E. coli*. Proteins were considered significantly changed in abundance if they had a two-sided rank-sum p-value < 0.05, after Benjamini-Hochberg correction for multiple testing, based on their fold-change compared to all other proteins (Fig 4). The underlying data for all panels, including GO annotations, can be found in S7 Table.
(PDF)

S5 Fig Genetic complementation in deletion mutants reverts caffeine-antagonisms to wild-type levels. (A) Caffeine shifts antibiotic MIC curves towards resistance. MIC curves (fitness versus antibiotic concentration) for the wild-type are shown for ciprofloxacin (left) and amoxicillin (right) with and without 55.5 µg/ml caffeine. The data shown is a snapshot of the checkerboard assays (Fig 5) at 0 and 55.5 µg/ml caffeine. Three individual replicates per experiment are shown (dots). The lines represent line-of-best fit a three-parameter logistic model (Materials and methods) using all replicates with and without caffeine. Dotted vertical and horizontal line represent 50% growth inhibition and corresponding concentration (IC_50_), respectively. (B) Caffeine MIC curves (growth versus caffeine concentration) of *E. coli* and *S.* Typhimurium wild-type strains. Average growth (OD_AUC_, dots) and standard deviation (error bars) over n biological replicates are shown. (C) Genetic complementation in deletion mutants reverts caffeine-MIC curves to wild-type levels. Upper panels correspond to ciprofloxacin/amoxicillin MIC curves with and without 55.5 µg/ml caffeine for the depicted deletion mutants, while the lower panels show the corresponding complementation. Data analysis was done as in panel a. The distance between the MIC curves is minimal in the deletion mutants (loss of antagonism), and re-established upon complementation. The data shown for the deletion mutants is a snapshot of the checkerboard assays (Fig 5) at 0 and 55.5 µg/ml caffeine. (D and E) Caffeine induces marRABp in a Rob-dependent manner. (D) Luminescence (RLU) profiles of marRABp basal activity (grey) and with increasing concentrations of caffeine (conc, S2 Table) over time are shown. Mean values of two biological replicates are shown. (E) Z-scores of caffeine-marRABp interaction showing its dependency on Rob. Lines are colored by strain and indicate mean Z-scores of two biological replicates (dots). (F) Deletion of *marA* does not affect the antagonism between ciprofloxacin and caffeine. Isobologram for caffeine-ciprofloxacin for *E. coli* Δ*marA* as done in Fig 5. (G) Complementation of *rob* in the Δ*rob* mutant re-enabled activation of marRABp transcriptional activity upon caffeine treatment (as measured by our luminescence reporter), as in the wild-type. Luminescence (RLU) profile over time for marRABp basal activity (water) and upon treatment with caffeine in 96 well-plates are shown. Average across 3 biological replicates is shown, error bars represent standard deviation. The underlying data for all panels can be found in S7 Table.
(PDF)

S1 Raw Images Original and uncropped minimally adjusted images of western and northern blots shown in Figs 4–6. (PDF)

S1 Table Bacterial strains, plasmids and oligonucleotides used in this study. (XLSX)

S2 Table Compound library with concentrations used for reporter screening. (XLSX)

S3 Table Compound screening results for wild-type and regulator mutants. OD_AUC_, LUX_AUC_ and LUX/OD measure for individual replicates of each compound-promoter pair, with indication whether values were removed due to poor growth.
(XLSX)

S4 Table Scores and Z-scores of individual replicates of each compound-promoter pair for wild-type and regulator mutants. (XLSX)

S5 Table Regulator contributions results and cross-validation outcome (statistical model): Coefficients β, B*, WT mean Z-scores, p-values and R^2^ values per compound-promoter pair. (XLSX)

S6 Table Analysed 2D TPP results for wild-type and Δ*rob* upon caffeine-treatment. (XLSX)

S7 Table Source data with all numeric values plotted in all main and supplementary figures. (XLSX)

S1 Data Raw data from compound screening - Growth (OD_600_) over time for individual replicates of each compound-promoter pair. (TXT)

S2 Data Raw data from compound screening - Luminescence (RLU) over time for individual replicates of each compound-promoter pair. (TXT)

S3 Data Raw data checkerboard assays - Growth (OD_600_) over time across concentrations for individual replicates of each pairwise drug combination from Figs 5, 6 and S5. (TXT)

S4 Data Raw data for MIC curves for complementation experiments - Growth (OD_600_) over time across concentrations for individual replicates of each mutant from S5 Fig. (TXT)
