# Supplementary figures and images for: Systematic screen uncovers regulator contributions to chemical cues in Escherichia coli
Source: PLoS Biol. 2025 Jul 22;23(7):e3003260. doi: 10.1371/journal.pbio.3003260 (PMC12282887; doi:10.1371/journal.pbio.3003260)

**A**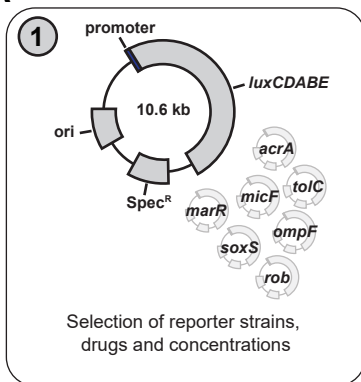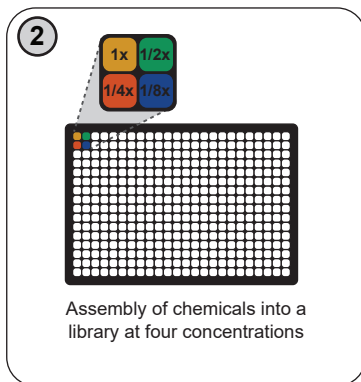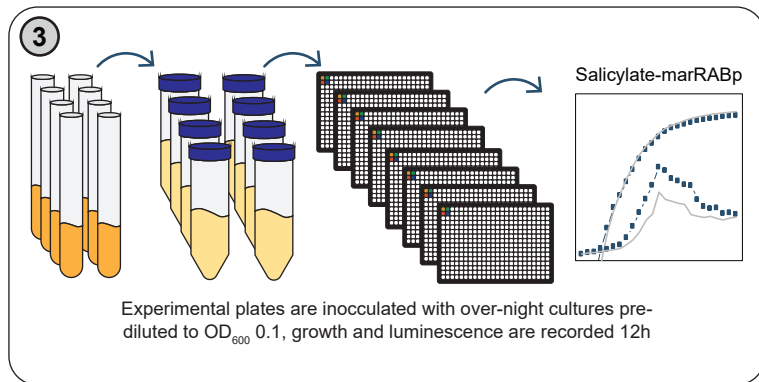**B**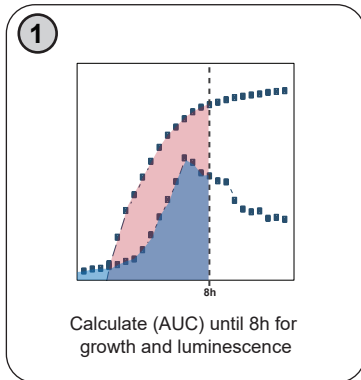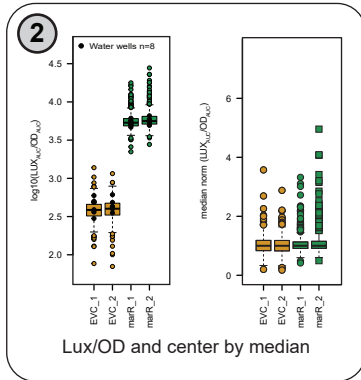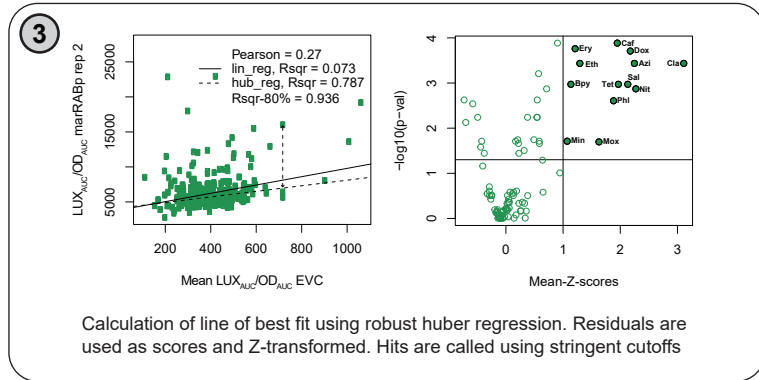**C**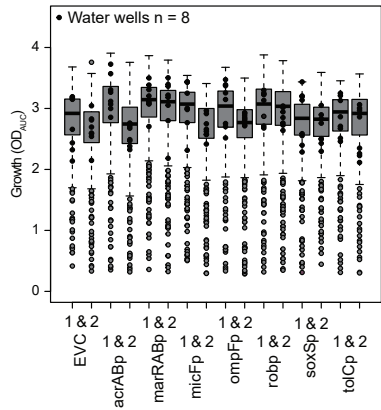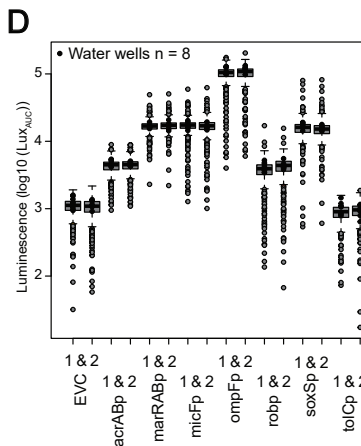**E**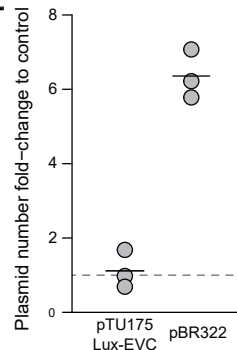**F**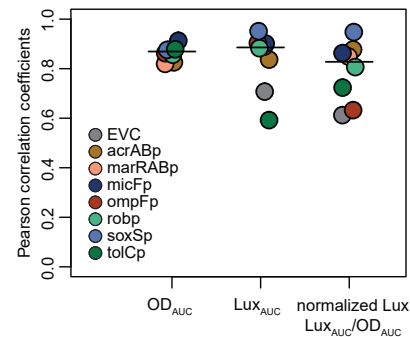

Supplement: S1 Fig — (A) Schematic screen workflow. Details described in Materials and methods. (A) Schematic of data processing. Details described in Materials and methods. (C) Boxplots of growth (ODAUC) across all reporters and replicates. Each boxplot represents a 384 well-plate (n = 384). Negative controls (water treatment) are displayed in black (n = 8 per strain). 1 and 2 refer to biological replicates. Center, upper and bottom lines represent 25th, 50th and 75th percentiles, whiskers extend to 1.5x inter-quartile range (IQR) and points beyond whiskers are represented individually. (D) Boxplots of luminescence (AUCLUX) data across all reporters and replicates. Each boxplot represents a 384 well-plate (n = 384). Negative controls (water treatment) are displayed in black (n = 8 per strain). 1 and 2 refer to biological replicates. Center, upper and bottom lines represent 25th, 50th and 75th percentiles, whiskers extend to 1.5x IQR and points beyond whiskers are represented individually. (E) Treatment with protein biosynthesis inhibitors does not affect copy number of pTU175 plasmids. Relative fold-change of pTU175-Lux-EVC and pBR322 after treatment with 2 µg/ml chloramphenicol compared to a negative control using qPCR. Three biological replicates are shown, and the line represents the mean. (F) Pearson replicate correlation of growth (ODAUC), luminescence (LUXAUC) and normalized luminescence (LUXAUC/ODAUC) between the duplicates of each strain. Line represents the mean replicate correlations for each variable. The underlying data for all panels can be found in S7 Table. (PDF) [file pbio.3003260.s001.pdf]

**A**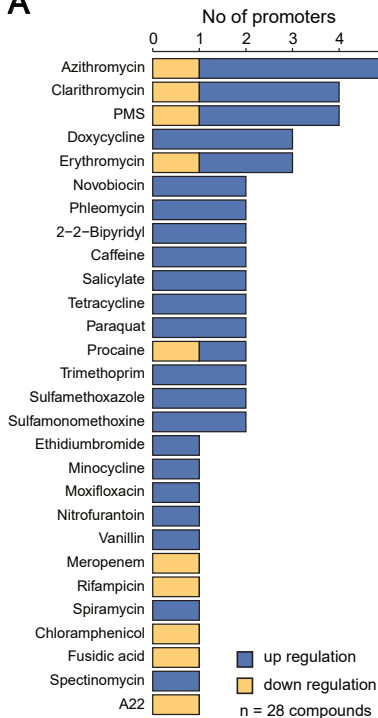**B**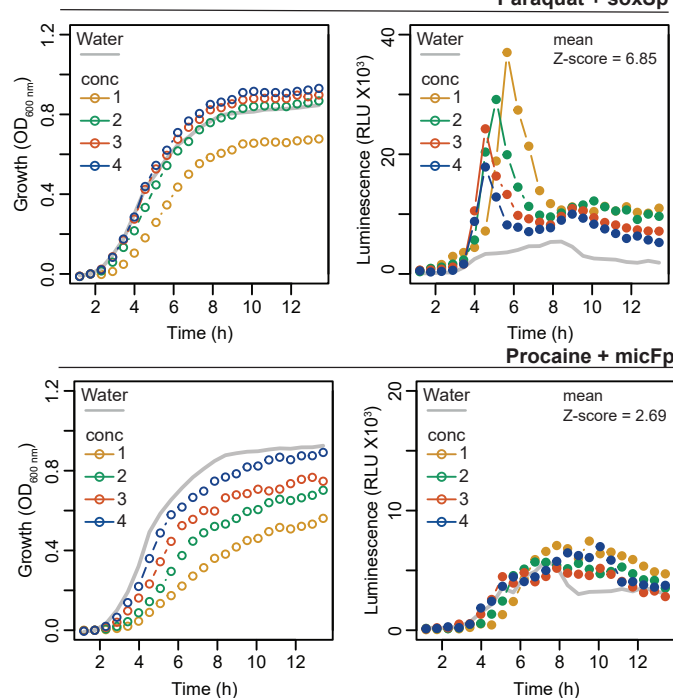**D**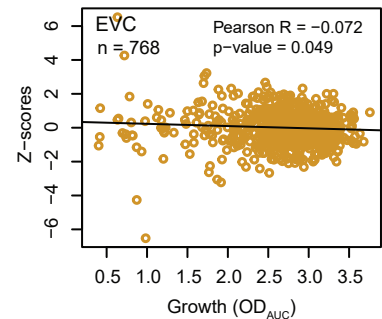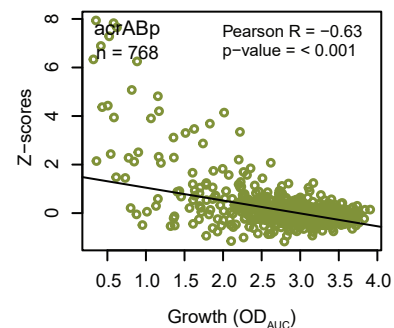**C**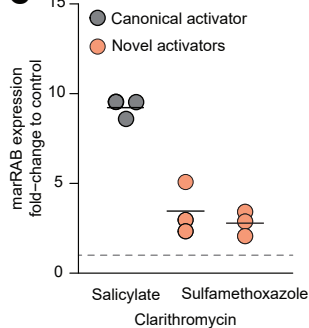**E**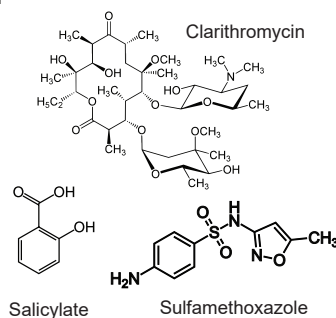**F**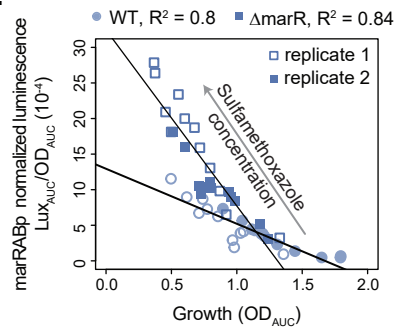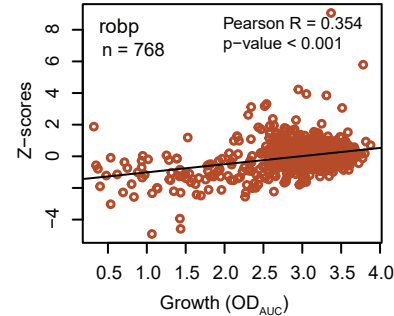

Supplement: S2 Fig — (A) General features of CPIs: number of CPIs per compound classified as up and down-regulation. (B) Previously known CPIs paraquat-soxSp [17] and procaine-micFp [31] are captured by our screening approach. Growth (OD600 nm) and luminescence (RLU) profiles over time for soxSp (top) and micFp (bottom) basal activity (grey) and with increasing concentrations of paraquat or procaine, respectively (conc, S2 Table) are shown. Mean values of two biological replicates are shown. (C) Clarithromycin and sulfamethoxazole are novel inducers of marRABp expression. RNA levels of marA after treatment with salicylate (positive control), clarithromycin and sulfamethoxazole. Data was double normalized to a non-treated control and to the house-keeping gene gyrA (Materials and methods). Three biological replicates are shown, and the line represents the mean. (D) Correlation between growth and promoter activity for EVC, acrABp and robp. Z-scores of all compound-EVC/acrABp/robp tested pairs including water across all 4 concentrations and 2 biological replicates (n) are plotted against growth (ODAUC). Pearson correlation coefficients (R) indicate no-, negative and positive correlation for EVC, acrABp and robp, respectively. Correlation p-value (double sided t test) shown. Linear relationships are illustrated by lines of best fit (Huber robust model). (E) Chemical structures of known and novel marRABp inducing compounds. (F) Induction of marRABp by sulfamethoxazole, as well as its negative correlation with growth, are independent of MarR. Luminescence profiles over growth were measured across a linear range of sulfamethoxazole concentrations from 0 µg/ml to 101.2 µg/ml in wild-type and ∆marR. Growth-normalized luminescence is plotted against growth for two independent biological replicates, and lines-of-best-fit (pooled replicates) are shown to highlight strong correlation between the two variables. The underlying data for all panels can be found in S7 Table. (PDF) [file pbio.3003260.s002.pdf]

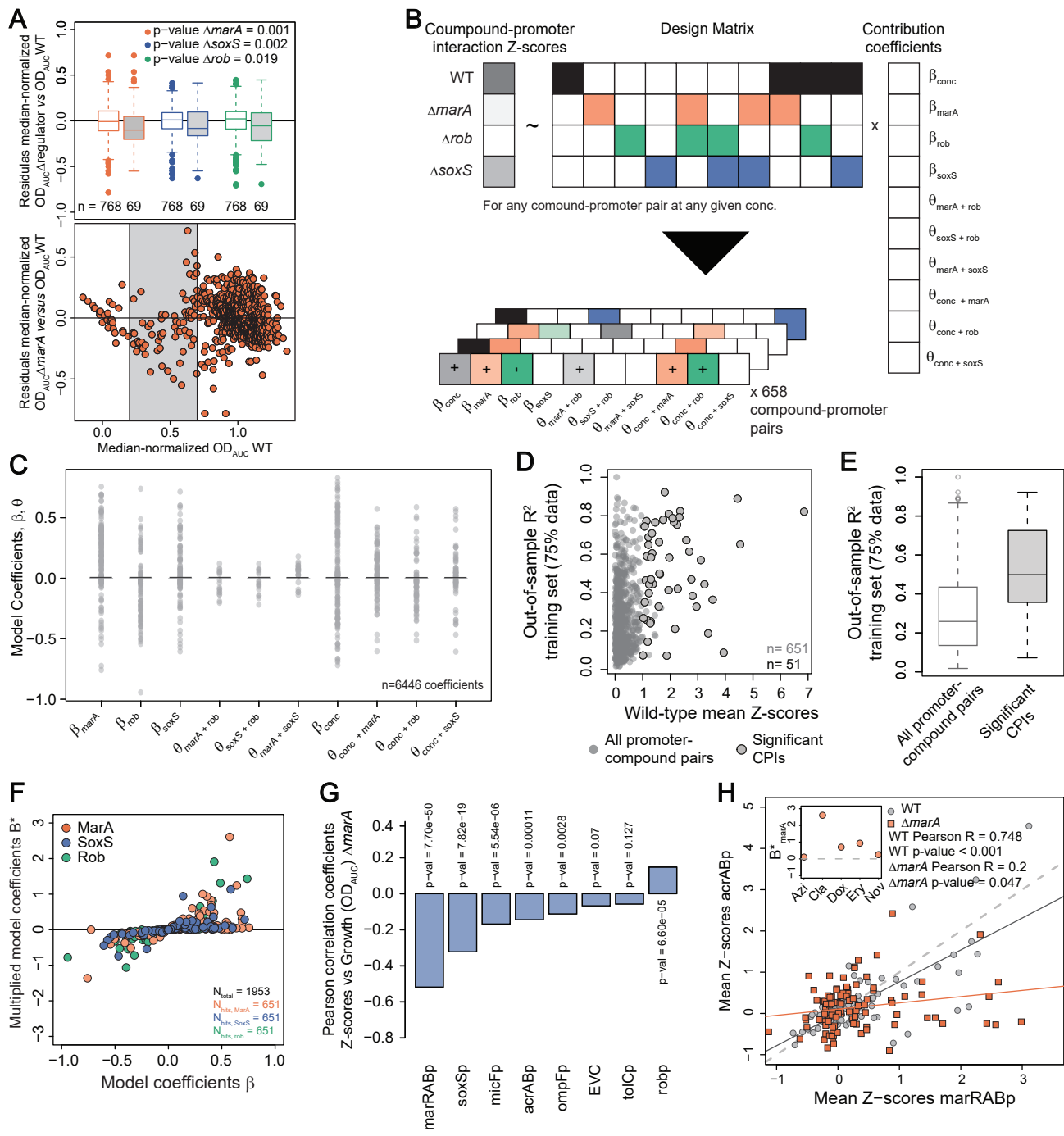

Supplement: S3 Fig — (A) Deletion of marA, soxS or rob sensitizes bacteria to several compounds at sub-inhibitory concentrations. Top: boxplots of the residuals of the lines-of-best-fit between growth of the regulator mutants and wild-type for all tested compound-promoter pairs including water across all 4 concentrations and 2 biological replicates (median normalized ODAUC within each strain). Only data from the promoterless control (EVC) is used. Negative residuals represent compound concentrations to which the regulator mutant is more sensitive than the wild-type. White boxes represent all pairs, and grey boxes correspond to the subset of pairs with 0.2 < wild-type median normalized ODAUC < 0.7, respectively. The number of data points is indicated below each box plot. Boxplots indicate 25th, 50th and 75th percentiles, and whiskers extend up to 1.5x the interquartile range (IQR) from the 25th and 75th percentiles. p-value from a one-sided statistical t test comparing full and subset residuals per mutant are shown. Bottom: Residuals of the lines-of-best-fit between growth (median normalized ODAUC) of ΔmarA and wild-type for all tested compound-promoter pairs across all 4 concentrations, including water and 2 biological replicates plotted against growth of the wild-type (median normalized ODAUC). Only data from the promoterless control (EVC) is used. Grey region corresponds to 0.2 < wild-type median normalized ODAUC < 0.7. (B) Schematic of the Lasso regression model to estimate regulator contributions to CPIs. Details described in Materials and methods. (C) Boxplots of contribution coefficients b and q grouped by name. Center, upper and bottom lines represent 25th, 50th and 75th percentiles, whiskers extend to 1.5x IQR and points beyond whiskers are represented individually. Due to the nature of the data – very sharply zero-centered – 25th, 50th and 75th overlap. (D) Scatterplot of out-of-sample R2 versus wild-type mean Z-scores for all compound-promoter pairs. CPIs are represented with [file pbio.3003260.s003.pdf]

**A**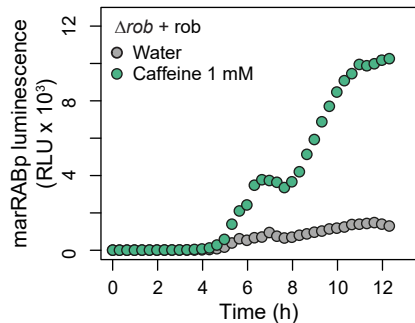**B**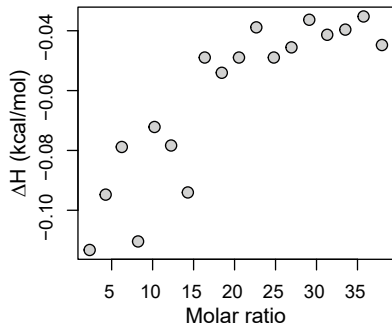**C**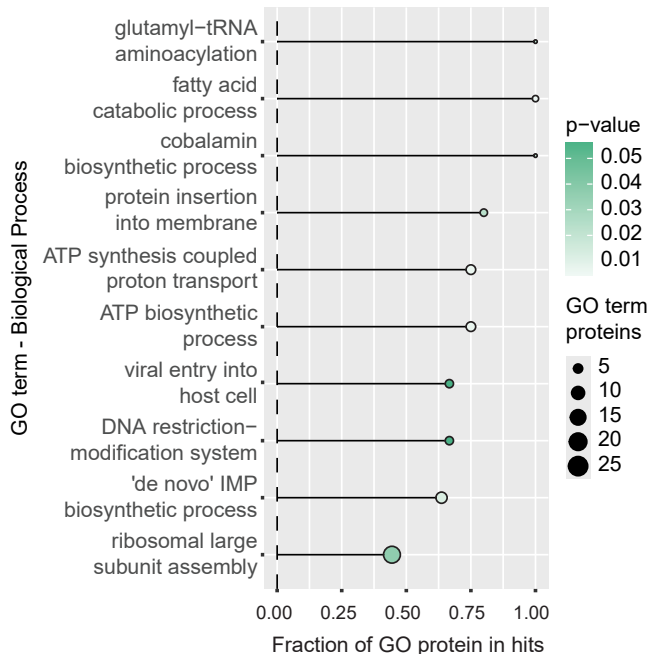

Supplement: S4 Fig — (A) Complementation of rob in the Δrob mutant re-enabled activation of micFp transcriptional activity upon caffeine treatment (as measured by our luminescence reporter), as in the wild-type. Luminescence (RLU) profile over time for micFp basal activity (water) and upon treatment with caffeine in 96 well-plates are shown. Average across 3 biological replicates is shown, error bars represent standard deviation (albeit very small, and therefore not visible). (B) Caffeine does not interact with Rob. The plot shows a binding isotherm representing the integrated heats (after baseline and dilution correction) over increasing molar ratio of caffeine-Rob, as obtained by ITC. (C) Gene Ontology enrichment analysis for protein abundance changes upon caffeine treatment in E. coli. Proteins were considered significantly changed in abundance if they had a two-sided rank-sum p-value < 0.05, after Benjamini-Hochberg correction for multiple testing, based on their fold-change compared to all other proteins (Fig 4). The underlying data for all panels, including GO annotations, can be found in S7 Table. (PDF) [file pbio.3003260.s004.pdf]

**A**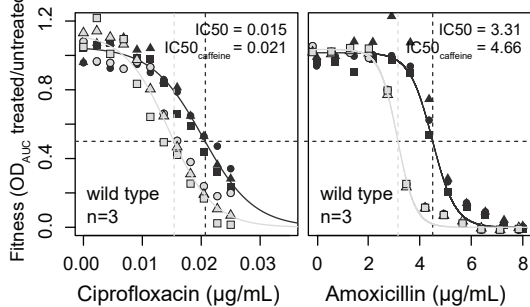**B**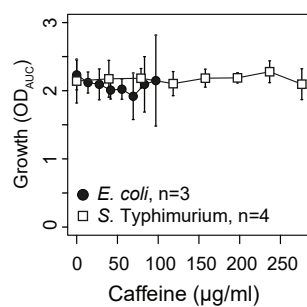**C**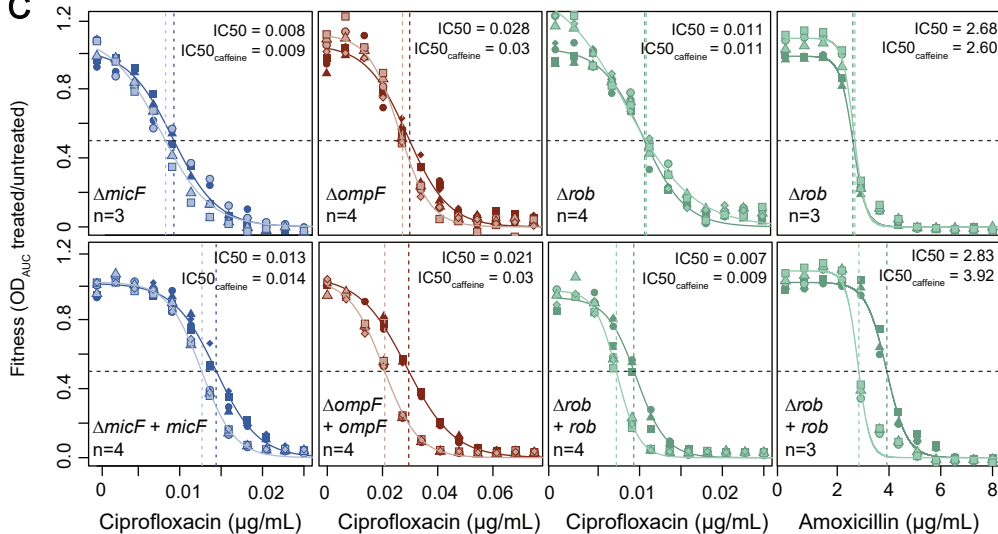**D**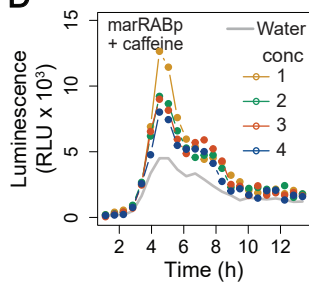**E**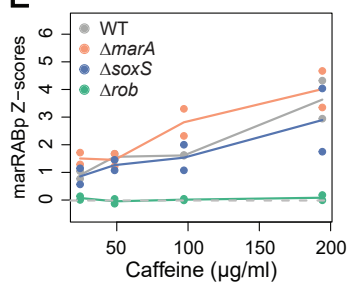**F**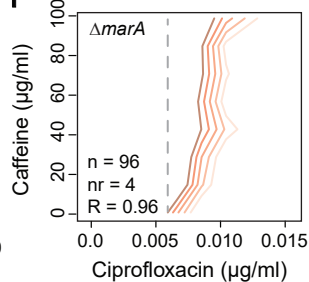**G**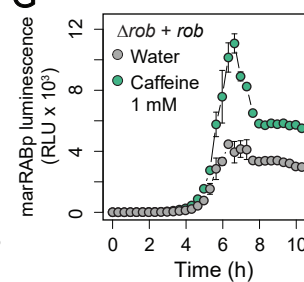

Supplement: S5 Fig — (A) Caffeine shifts antibiotic MIC curves towards resistance. MIC curves (fitness versus antibiotic concentration) for the wild-type are shown for ciprofloxacin (left) and amoxicillin (right) with and without 55.5 µg/ml caffeine. The data shown is a snapshot of the checkerboard assays (Fig 5) at 0 and 55.5 µg/ml caffeine. Three individual replicates per experiment are shown (dots). The lines represent line-of-best fit a three-parameter logistic model (Materials and methods) using all replicates with and without caffeine. Dotted vertical and horizontal line represent 50% growth inhibition and corresponding concentration (IC50), respectively. (B) Caffeine MIC curves (growth versus caffeine concentration) of E. coli and S. Typhimurium wild-type strains. Average growth (ODAUC, dots) and standard deviation (error bars) over n biological replicates are shown. (C) Genetic complementation in deletion mutants reverts caffeine-MIC curves to wild-type levels. Upper panels correspond to ciprofloxacin/amoxicillin MIC curves with and without 55.5 µg/ml caffeine for the depicted deletion mutants, while the lower panels show the corresponding complementation. Data analysis was done as in panel a. The distance between the MIC curves is minimal in the deletion mutants (loss of antagonism), and re-established upon complementation. The data shown for the deletion mutants is a snapshot of the checkerboard assays (Fig 5) at 0 and 55.5 µg/ml caffeine. (D and E) Caffeine induces marRABp in a Rob-dependent manner. (D) Luminescence (RLU) profiles of marRABp basal activity (grey) and with increasing concentrations of caffeine (conc, S2 Table) over time are shown. Mean values of two biological replicates are shown. (E) Z-scores of caffeine-marRABp interaction showing its dependency on Rob. Lines are colored by strain and indicate mean Z-scores of two biological replicates (dots). (F) Deletion of marA does not affect the antagonism between ciprofloxacin and caffeine. Isobologram for caffeine-ci [file pbio.3003260.s005.pdf]
