# Supplementary material for: Systematic screen uncovers regulator contributions to chemical cues in Escherichia coli
Source: PLoS Biol. 2025 Jul 22;23(7):e3003260. doi: 10.1371/journal.pbio.3003260 (PMC12282887; doi:10.1371/journal.pbio.3003260)

Fig 4a – MicF signal

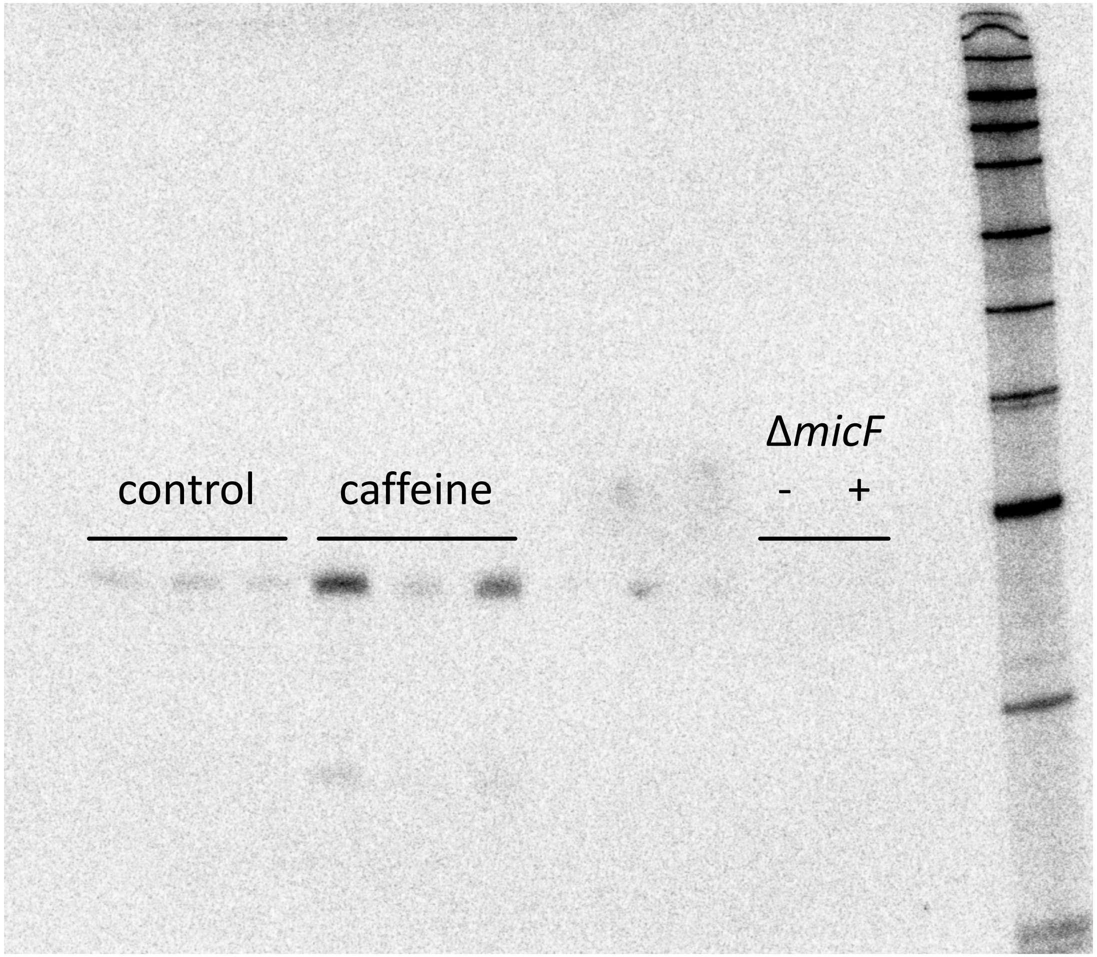

Fig 4a – 5S rRNA signal

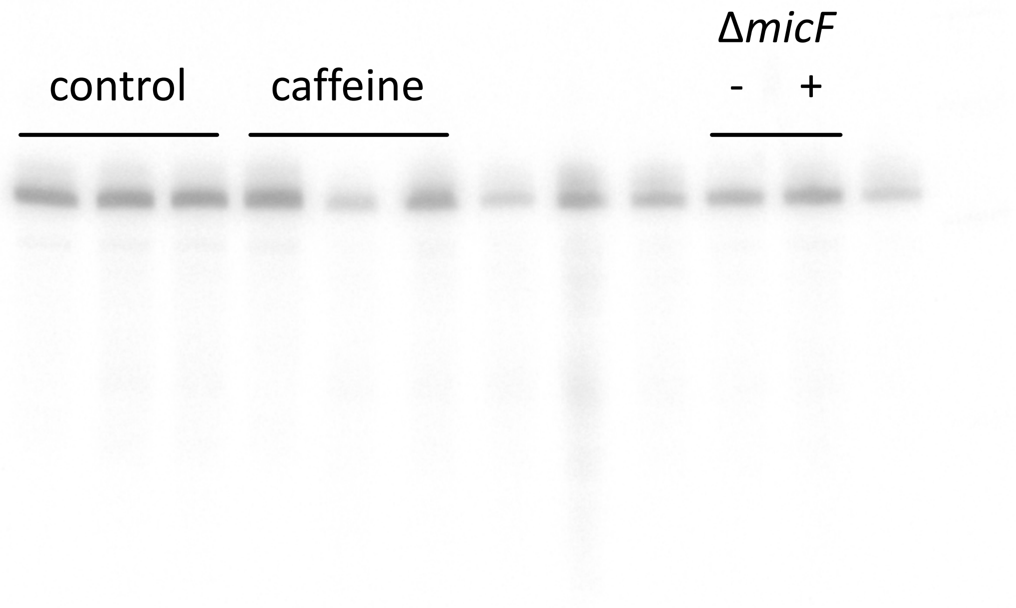

Fig 5a – OmpF signal

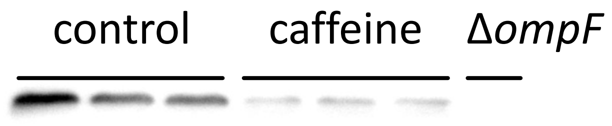

Fig 5a – RecA signal

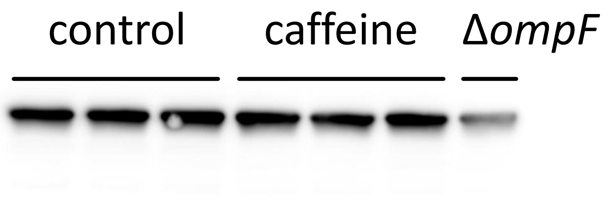

Fig 6c – OmpF signal

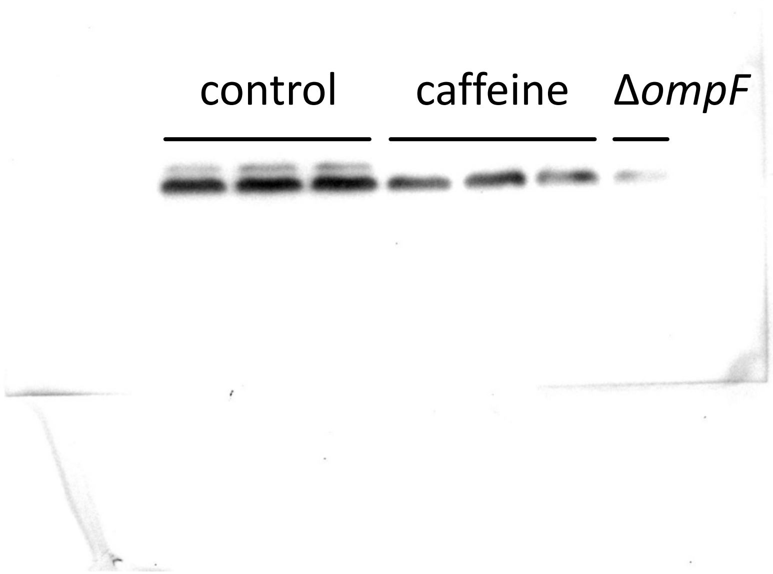

Fig 6c – RecA signal

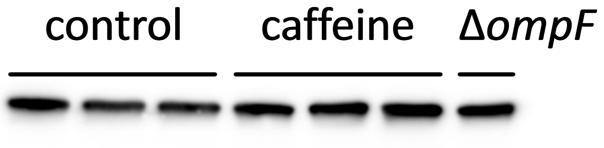

Supplement: S1 Raw Images — (PDF) [file pbio.3003260.s006.pdf]
